# Acid-assisted water loss in perrhenate-catalysed deoxydehydration: an agent-assisted reaction-network search with microkinetic modelling

**DOI:** 10.1039/d6sc06865k

**Published:** 2026-10-27

**Authors:** Joshua M. Sims, Jeremy N. Harvey

**Affiliations:** a Department of Chemistry, KU Leuven Celestijnenlaan 200F B-3001 Leuven Belgium joshua.sims@kuleuven.be

## Abstract

Perrhenate is the cheapest and most robust deoxydehydration (DODH) catalyst. Two decades of reports show that protic and acidic environments make it work, and no study has located where the proton acts. We answer that question by providing a predicted mechanism that is consistent with observed kinetics. The authors set the mechanistic hypotheses, and an AI agent located the corresponding transition states, which the authors then validated; solvent participation was resolved shell by shell, including its complex temperature dependence; and the free energy surface was converted into rates *via* microkinetic simulation on coupled-cluster-anchored energies. The predicted mechanism is proton-gated at its water-loss steps: a proton converts Re–OH into water, which leaves, thereby unlocking both the diol ring closure and alcohol reduction steps. This yields an increase in the predicted rate by eight orders of magnitude over the best proton-free chemistry we could locate. Compared like-for-like with the batch experiments, the model falls within the 31.5–34.0 kcal mol^−1^ window derived from reported turnover.

## Abstract 2

An agentic-AI assisted investigation has resulted in proposing a novel mechanism for perrhenate DODH that is consistent with experimental turnover frequencies.

## Graphical abstract

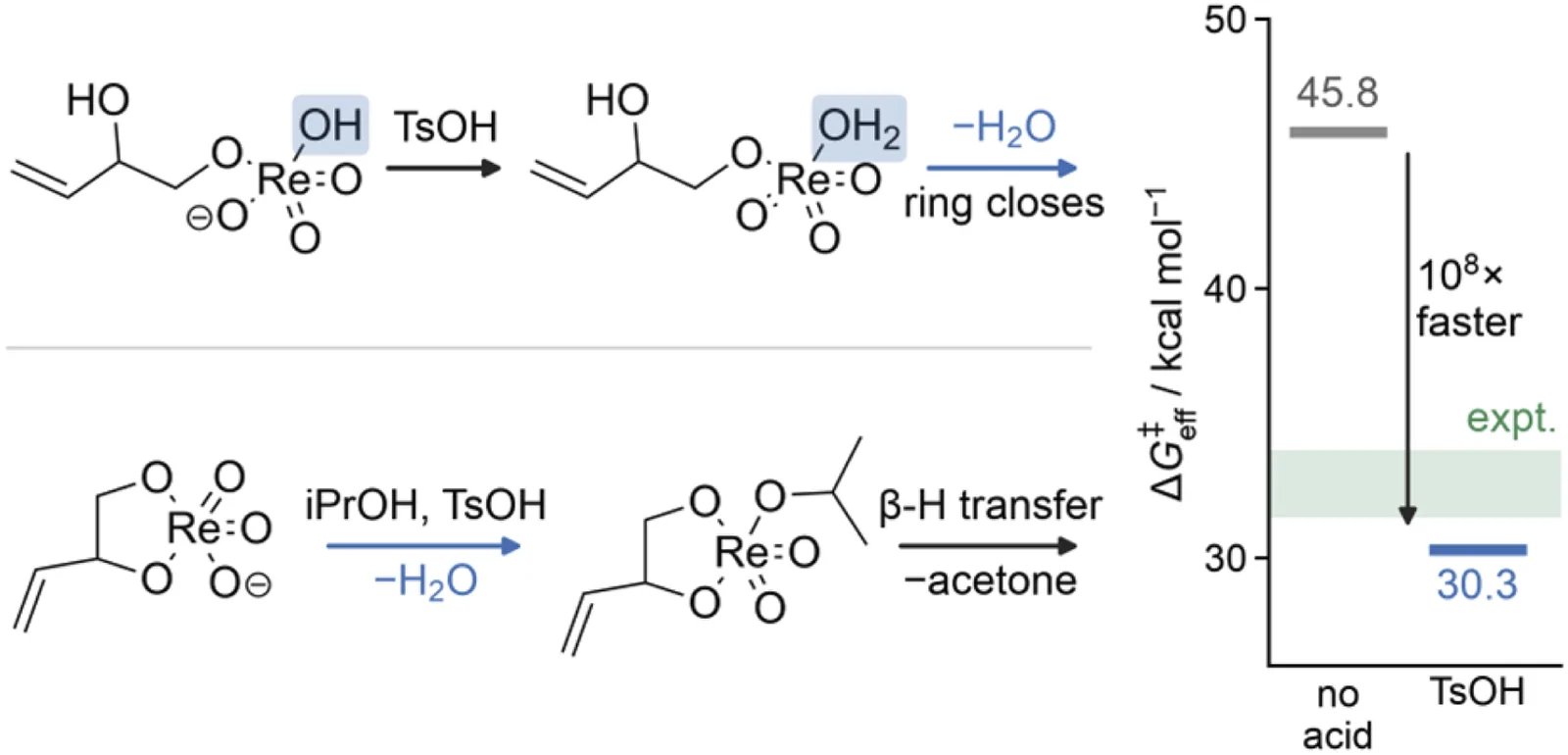

## 1. Introduction

Deoxydehydration converts a vicinal diol into an olefin and water in a single catalytic step, and for that reason it sits at the centre of most schemes for defunctionalising biomass-derived polyols.^1–4^ Among its catalysts, simple perrhenate salts occupy a peculiar position. They are the cheapest and most robust catalysts in the rhenium family; they tolerate air and water, and they turn over glycols, glycerol, and sugar-derived diols at 145–175 °C for hours to days, with alcohol or sulfite reductants.^3,5–8^ The mechanistic canon for DODH was built on methyltrioxorhenium (MTO) and Cp*ReO_3_,^9–13^ neutral platforms whose speciation, resting states and kinetics differ from those of a bare anion, and a recent kinetic study of Cp*ReO_3_ found that even for the oldest DODH catalyst the identity of the active species is still unresolved.^14^ Re(vii) diolates have been shown to form readily from MTO and diols^12^ or epoxides, and triphenylphosphine can be shown to reduce them at room temperature to MTO and the olefin.^15^ Generally speaking, unless the reductant is particularly strong, condensation has been argued to occur first, followed by reduction.^6,10,15^ For ReO_4_^−^ itself, previous computational studies have consisted of static density–functional profiles in implicit solvent,^16,17^ and the experimental record includes an unexplained observation: protic and acidic environments accelerate the reaction. Pyridinium and protic ammonium perrhenates outperform their alkali analogues,^17^ added Brønsted acid accelerates Re-catalysed DODH of a sugar-acid substrate,^18^ pyridinium salts outperform quaternary-ammonium ones,^19^ and heterogenised perrhenate responds to the support and its acidity.^20,21^ The sharpest of these observations is quantitative: with a dirhenium carbonyl precursor that oxidises *in situ* and 3-octanol as reductant, a diol that does not react at all at 155 °C in 5 h goes to complete conversion in 2.5 h once 5 mol% of tosylic acid is present, and 2 mol% leads to a 15 °C reduction in temperature with a tenfold increase in rate.^22^ No study has located where the proton enters the cycle or what it does there.

Perrhenate DODH presents particular challenges: the reaction could proceed along reduction-first or condensation-first paths; various numbers of solvent alcohol molecules can be involved; several protonation states need to be accounted for, as well as possible binuclear channels. In this work, we have tackled these challenges by mapping the free-energy profile across a broad range of catalyst structures and reaction-step orders using an agentic, AI-driven search built on automated transition-state localisation and human validation. This approach is similar to the lineage of automated reaction-network exploration methods and to agent-operated chemistry methods more broadly.^23–27^ Among agentic approaches, the present work is to our knowledge the first to generate a complete homogeneous catalytic cycle. This adds a new contribution to automated reaction path searching, which is already used in catalysis.^28^ Converting a computed free-energy surface into rates through an explicit kinetic model, rather than reading a rate-determining span off the profile, is increasingly recognised as necessary for quantitative comparison with experiment.^29,30^ While microkinetic modelling has been used in DODH mechanistic studies before, this has been only for heterogeneous rhenium and polyoxometalate catalysts;^31,32^ for the homogeneous perrhenate system, computational studies have never gone beyond free-energy profiles. We do so based on composite free energies anchored to DLPNO-CCSD(T)/CBS electronic energies. The resulting predicted kinetics are compared to experimental results. Building the mechanism as a ladder of increasingly complete models, we find that the two-cycle prior proposals reduce to a mechanism that is predicted not to turn over at all under experimental conditions. Including a second equivalent of the alcohol reductant improves the description of the reduction chemistry, yet leaves the best proton-free predicted reaction rate roughly seven orders of magnitude lower than experiment. The presence of acid, in this instance tosylic acid, acts within the established condensation-first sequence: it protonates labile Re–OH groups, which then leave as water. The simulated kinetics of this acid-assisted mechanism land in the experimental window. Furthermore, this mechanism is not specific to tosylic acid: applying the same chemistry with weaker proton donors, such as those sometimes used as counterions for perrhenate, yields similar results.

## 2. Computational methods

Geometries and frequencies were calculated at the B3LYP-D3(BJ)^33,34^/def2-SVP^35^ level with the Stuttgart–Dresden effective core potential on rhenium,^36^ with PCM to account for solvation,^37^ using Gaussian 16.^38^ Transition states were validated by possessing exactly one imaginary frequency, along a mode that corresponds to the expected reaction, as well as an intrinsic reaction coordinate calculation leading to the correct reactants and products.^39^ Key transition states are shown in Fig. S1. Free energies were computed using the quasi-harmonic approximation (GoodVibes,^40^ with all vibrational frequencies below 50 cm^−1^ raised to 50 cm^−1^),^41^ with the neat concentration of the solvent taken into account (this correction can occasionally cause low-barrier transition states to be plotted below an adjacent intermediate; these situations are plotted as such). Tunnelling effects were computed using Wigner^42^/Skodje–Truhlar^43^ tunnelling corrections, reported at 413.15 K. All reported energies were corrected by doing single-point calculations at the PBE0-D3(BJ)^44^/def2-TZVP level, with DLPNO-CCSD(T)^45^/CBS (def2-TZVPP→QZVPP;^46^ ORCA^47^) anchors on all rate-relevant saddles (Table S1). Conformers of all structures were located by subjecting structures to a metadynamics-based conformer method (Section S3).

The microkinetic model uses transition-state-theory rate constants for each elementary step in the coupled reaction networks shown in Fig. 3 and 4 (Section S6.1 and Table S5). Apparent energy barriers, resting states, reaction orders, and the Campbell degree of rate control,^48^*X*_RC,*i*_ = (∂ln *r*/∂ln *k*_*i*_) at fixed *k*_*i*_, which indicates how the overall rate responds to the free energy of transition state *i* (the sum of *X*_RC,*i*_ values is one; *X*_RC_ = 1 identifies a rate-determining step), were derived from the quasi-steady-state solution of the network. Concentration–time profiles were obtained by integrating the same network as a batch reactor (Fig. S5). To keep the discussion short, in the text below, we convert observed turnover rates from microkinetic modelling into effective free energy barriers by inverting the Eyring equation, and report these effective free energy barriers.

For the agentic search aspect of the calculations: a large-language-model agent (Claude Code) was used to run the full proposal–calculation–triage loop, proposing elementary steps, building and dispatching the electronic-structure jobs, triaging the returns evaluated against a set of predefined acceptance factors, and maintaining the provenance chain, all under human-defined criteria it cannot relax. The mechanistic hypotheses were set by the authors (Scheme 1). For each hypothesis, the agent takes the structures of the reagents and/or products as multimodal input, for example a drawn reaction scheme or an image, or simply a written description, builds three-dimensional endpoints from optimised minima with consistent atom ordering, and locates and validates the transition states itself (growing-string search with g-xTB, then transition-state optimisation, frequencies and IRC at the level given above; Section S1), as well as any intermediates not specified by the authors but deducible from the route they describe. In particular, a skill for locating and validating transition states has been developed and is available at https://github.com/joshazerty/chem-skills, enabling the protocol to be rerun on other systems. All minima and transition states located through the agentic protocol were verified and validated by a human.

**Scheme 1:**
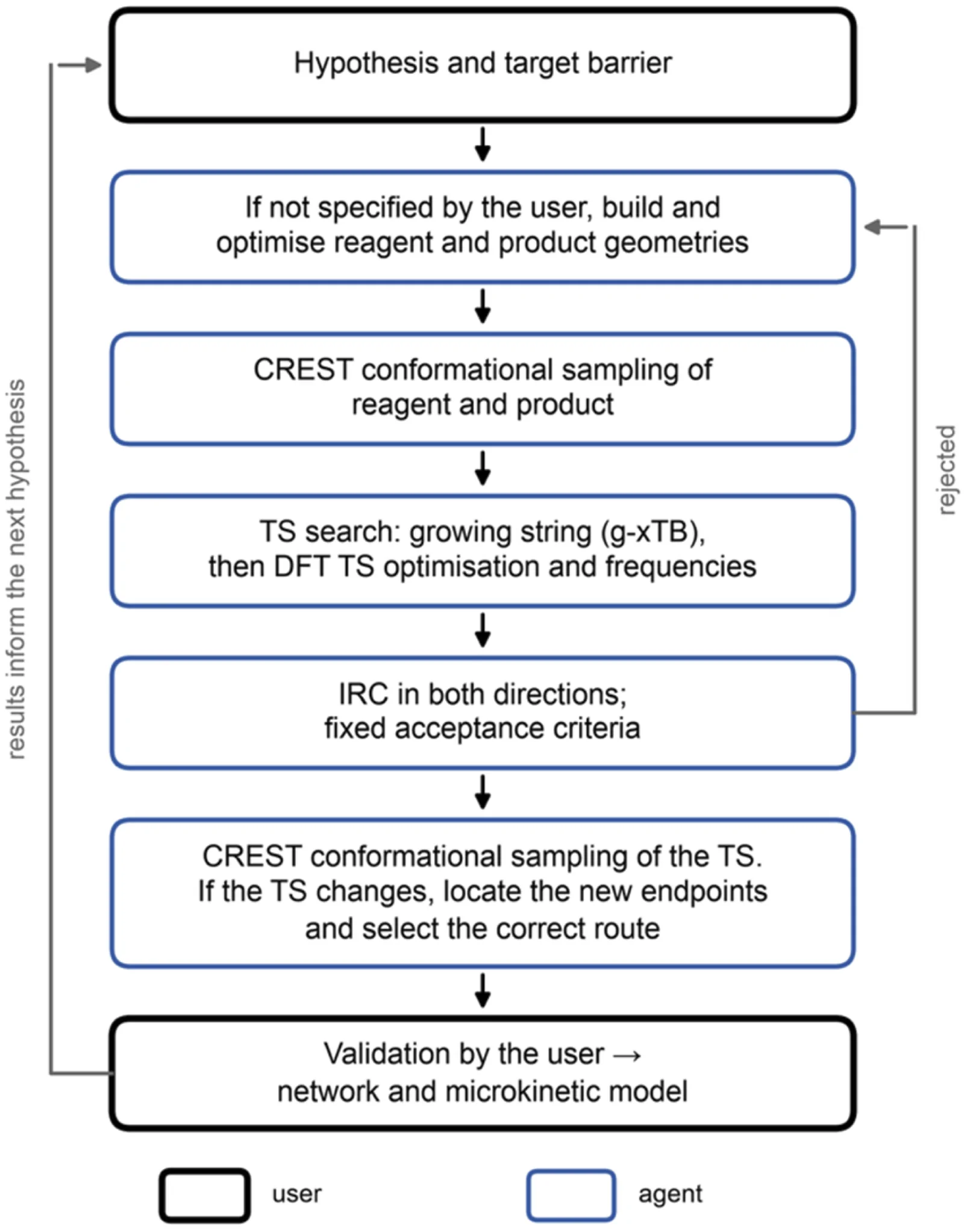
Workflow of the agent-assisted network search. The steps carried out by the user are shown as black boxes and those carried out by the agent as blue boxes.

## 3. Results and discussion

### 3.1 Proton-free mechanisms

Our experimental reference point is the DODH of a substrate such as but-3-ene-1,2-diol to butadiene, using a secondary alcohol such as 3-pentanol as the solvent and reductant. In our computational model, we take but-3-ene-1,2-diol as substrate, isopropyl alcohol as co-reactant, and pentanol as a continuum solvent model, with bare ReO_4_^−^ as catalyst and *p*-toluenesulfonic acid (TsOH) available as a co-catalyst, at the experimental operating temperature of 413 K. Our choice was motivated by practical relevance (butadiene is a volatile commodity target) and reactivity: the vinyl group provides allylic conjugation in the retro-[3 + 2] transition state, thereby lowering the extrusion barrier (by 6.4 kcal mol^−1^ in our calculations relative to that obtained with propane-1,2-diol). The retained network comprises 111 stationary points spanning Re(vii) and Re(v), including the idealised reduction-first and condensation-first pathways (Fig. 1), together with mono- and bis-isopropoxide reduction chemistry, four- and six-centre-membered-ring variants of every proton transfer TS, and acid-assisted pathways (the search protocol is in Section S1, and S3 reports the conformational-sampling campaign). All energies below are Δ*G* at 413 K with respect to the bare-perrhenate resting state 1 unless stated otherwise.

**Fig. 1:**
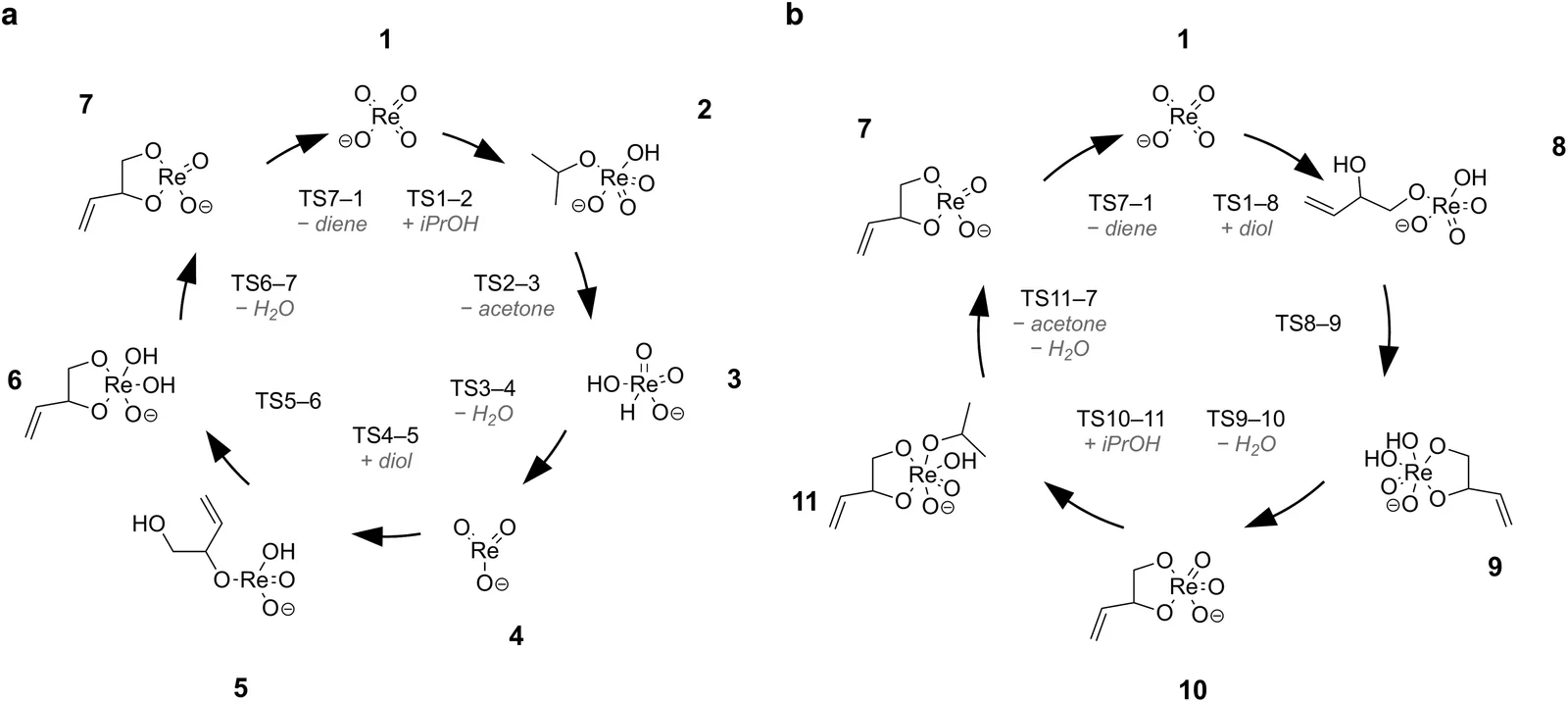
The two classical DODH cycles for bare perrhenate, acid-free: (a) reduction-first (RC), through the single-alcohol hydride reduction; (b) condensation-first (CR). The cycles share the resting state 1, the Re(v) diolate 7 and the cycloreversion TS7-1.

Fig. 1 shows the classical reaction mechanisms which dominate the literature. The reduction-condensation mechanism (Fig. 1a) proceeds *via* initial reduction of the catalyst by the solvent to yield 4. The diol then condenses onto the catalyst (in two steps) to yield 7 before an oxidative extrusion step regenerates the catalyst and liberates the desired alkene product. The condensation-reduction mechanism in Fig. 1b reverses the order: first, the two-step condensation of the diol to yield 10; then, the reduction of the catalyst by a solvent molecule; and finally, the same extrusion step from 7 as in the reduction-first mechanism.

With the rhenium atom described by the SDD effective core potential and valence basis set, small basis sets for the other atoms produce significant systematic errors, with ligands being typically very overbound towards the rhenium centre. For example, using the def2-SVP basis set, isopropanol overbinding is found, with the counterpoise correction denoting a basis-set superposition error of 8.0 kcal mol^−1^ in some cases. This is much improved when final energies are computed with a larger basis set, such as def2-TZVP, as in our study. Furthermore, the predicted cycloreversion barrier from species 7 scatters over 27.7 kcal mol^−1^ across the thirteen functionals we benchmarked on it, from +1.2 with r2SCAN-D4 to +28.9 with M06-2X. Our coupled-cluster benchmark value of +17.2 is near the middle of this range; the thirteen functionals are identified, and the full benchmark data given, in Section S2 and Table S2. The scatter is specific to this step type: the same thirteen functionals agree to within 3.7 kcal mol^−1^ on the hydride elimination step. The high sensitivity of the barrier height to the functional used is largely a dependence on the fraction of exact exchange, see Section S2, Fig. S2 and Table S3. This is not unusual when dealing with changes in oxidation state at a transition metal and to other changes in degrees of electronic delocalisation.^49^ The functionals we use span a very wide range of exact exchange admixtures; realistically, those with a moderate degree of exact exchange might be expected to be most reliable and that is indeed the case upon comparison to CCSD(T). Furthermore, single-reference diagnostics^50^ suggest that the intermediates and TSs studied here have almost exclusively single-reference character, supporting the use of DLPNO-CCSD(T)/CBS as a benchmark (Section S2, Fig. S2 and Table S3).

We located TSs for every genuine proton transfer twice: as a direct four-centre transfer within the complex, and as an alcohol-relayed six-centre ring in which a spectator isopropanol accepts the proton at one end of the transition state and delivers it at the other. Neither form wins everywhere, because the relay is an enthalpy-for-entropy trade (Fig. S3). The expanded ring leads to lower enthalpy (a hydrogen-bonded six-membered ring in place of a strained four-centre geometry, worth 9.8 to 28.6 kcal mol^−1^ across the eight pairs we considered). It pays for it by recruiting one solvent molecule from the bulk and holding it in the transition state. The resulting entropic price is nearly invariant across all eight rings, −26 to −30 cal mol^−1^ K^−1^, which is the ordinary association entropy of binding one molecule, indifferent to which bond is being shuffled (in reality this should not be truly constant, as the PCM correction should be temperature dependent; however PCM implementations are parametrised for only one value of *T*). The free-energy benefit of a relay mechanism decays with temperature at the same slope of 2.8 kcal mol^−1^ per 100 K (Fig. S3). Four of the eight pairs cross over between 373 and 487 K, a range that overlaps the 363–443 K window in which this chemistry is run (Table S4 and Fig. S3), and the model uses the lower of the two forms at 413 K. Accounting for active participation of solvent molecules does not, however, lower the barriers on all types of transition states: hydride transfer and cycloreversion steps are not helped and remain possible rate-limiting steps. A further shell of solvent, added outside the ring, pushes the other way again: two explicit solvent molecules relaxed onto the gating condensation saddles raise their free energy by up to +7 kcal mol^−1^, because those transition states have fewer free hydrogen-bond donors to satisfy than the reactants they came from (Section S5.3). With the solvent treated consistently at every proton transfer, we can now ask whether the classical cycles of Fig. 1 are fast enough to account for the observed chemistry.

Even with every proton transfer treated in its optimum relayed form, both classical cycles (Fig. 1) involve high-energy intermediates and transition states (Fig. 2). On the converged surface, the RC reduction is gated by its β-H transfer (Fig. 2a), either through the discrete rhenium hydride 3, whose reductive elimination of water (TS3-4) sits at +62.9 kcal mol^−1^, or hydride-free, the isopropoxide C–H migrating to an oxo ligand to give the dihydroxide 14 through TS2-14, at +66.8. The acid-free condensation-reduction route is gated by the anionic β-hydride transfer TS11-7 at +63.2 kcal mol^−1^, and the hydrogen-bond-stabilised variant raises the barrier (Section S4). At 413 K (typical operating temperature), these barriers correspond to predicted rates more than ten orders of magnitude below the observed rate (see Section S6.5).

**Fig. 2:**
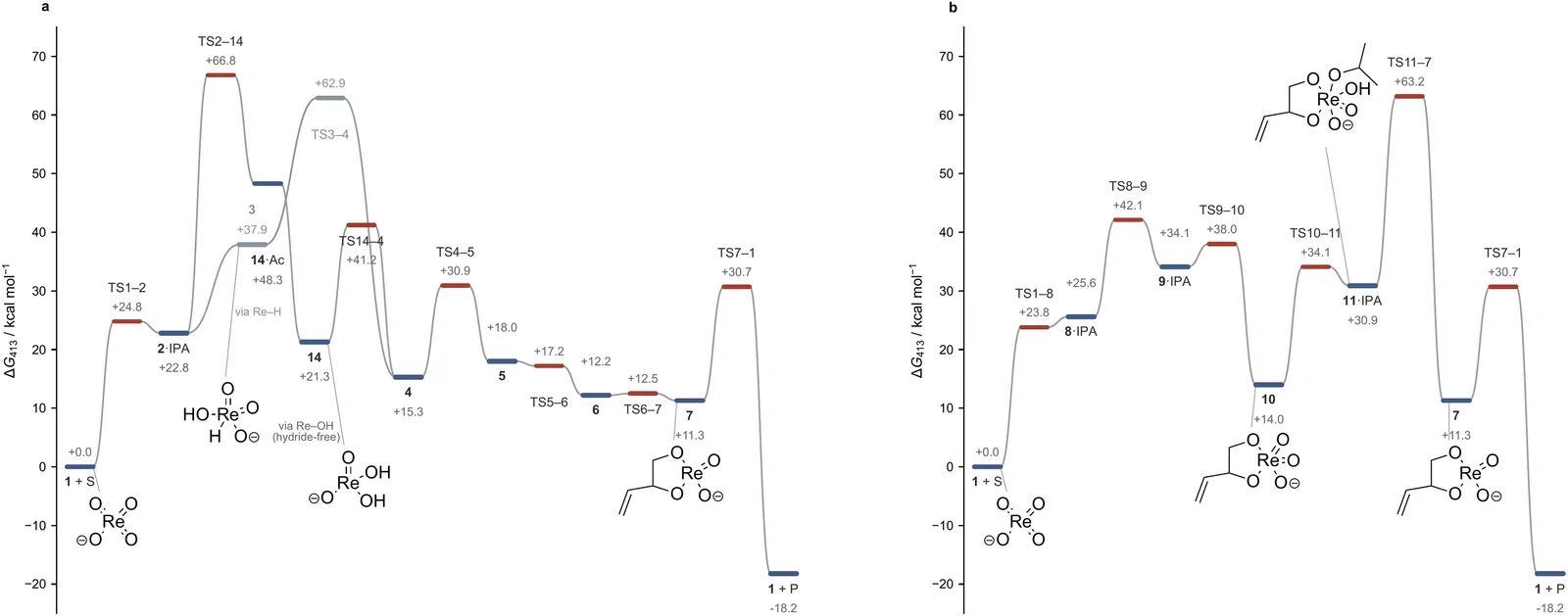
Free-energy profiles of the classical mechanisms, *vs.*1: (a) reduction-first, with both single-alcohol reduction legs drawn between 2·IPA and 4 – *via* the discrete hydride 3 (grey) and hydride-free *via* the dihydroxide 14 (TS2-14). (b) Condensation-first. Submerged relay saddles are drawn at their computed values. *S* is the diol substrate, and *P* is the alkene product.

The reference mechanisms of Fig. 1 can be substantially improved by including coordination of an additional solvent molecule onto 2 immediately before the reduction step, forming the bis-isopropoxide 12. The highest calculated barrier drops as a result of this change from +62.9 to +48.2 kcal mol^−1^ (Fig. 3). In this case, kinetic modelling provides a different picture from a naïve reading of the free energy profile. Read statically, Fig. 3 shows β-hydride transfer saddle TS12-13 as the potential rate-limiting step. Microkinetic modelling of the network shown in Fig. 3 returns an apparent Δ*G*^‡^_eff_ of 45.8 kcal mol^−1^ at 413 K and places essentially all rate control on the second condensation of a solvent molecule, TS2-12 (*X*_RC_ = 0.98). With an effective free energy barrier of 45.8 kcal mol^−1^, this revised acid-free network is still predicted to turn over seven orders of magnitude slower than is observed experimentally, implying an error of 12.8 kcal mol^−1^.

**Fig. 3:**
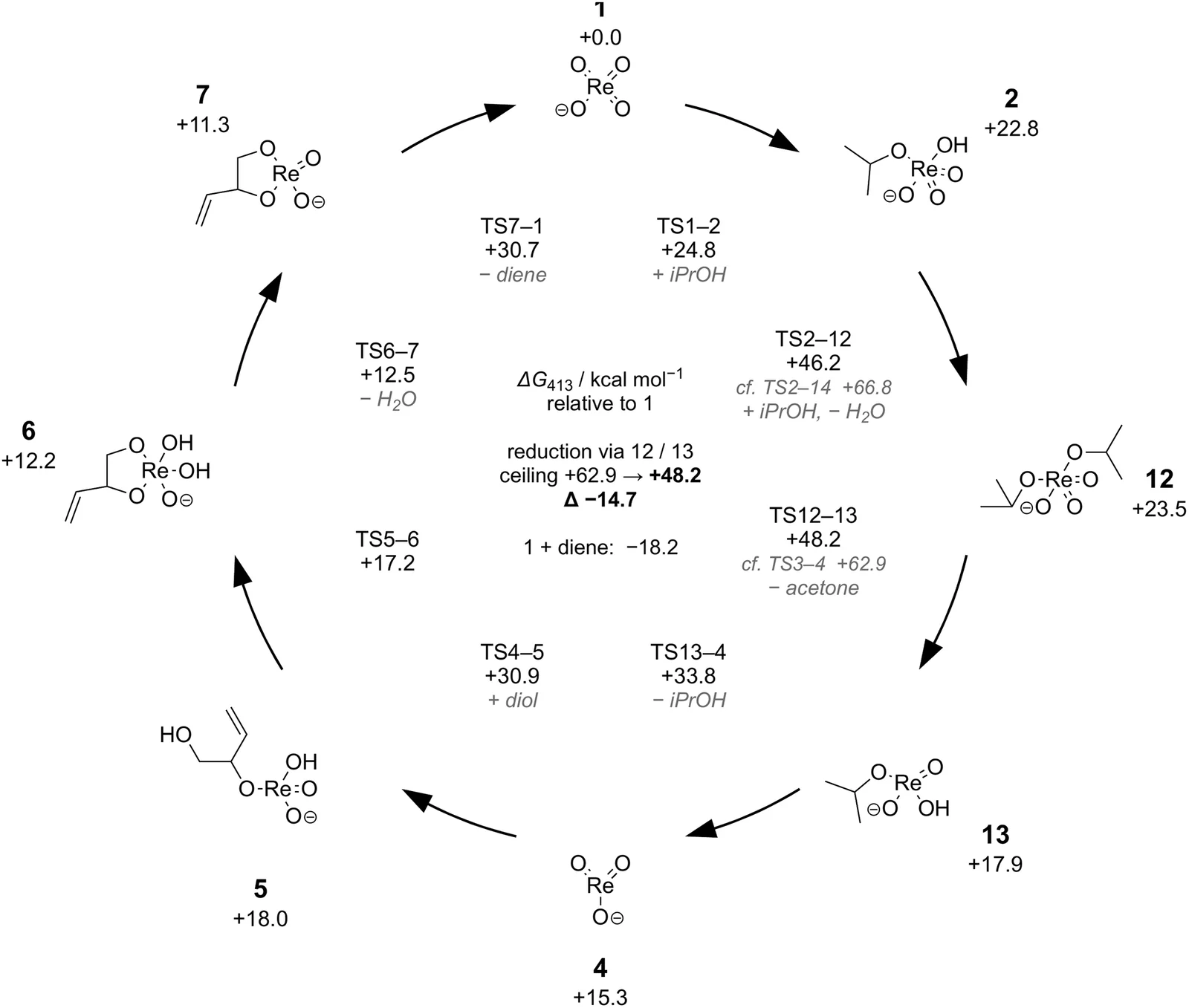
The reduction-first cycle with a second isopropanol: reduction through the bis-isopropoxide 12 and the β-hydride transfer saddle TS12-13, avoiding the discrete hydride. Free energies and free energy barriers are indicated in kcal mol^−1^, as well as the key differences with the classical reduction-condensation mechanism.

### 3.2 Acid-assisted mechanism

The missing ingredient, as is already known from experimental work, is a proton. It is often thought that the proton would act by protonating an oxygen atom in the complex, thereby facilitating one or more condensation steps. Here, the proton acts at a different stage by enabling an early water-loss step. Plain proton transfers or relays do nothing useful. Modelling TsOH as an explicit molecule on the limiting condensation saddle traps the substrate as a stable, unproductive ion pair (binding −9.3 kcal mol^−1^) in which the acid grips the alkoxide rather than advancing it (Section S5); and adding explicit outer-shell pentanol to the same saddles raises the barriers, exactly as it did on the acid-free condensations above (Section S5.3).

However, in the condensation-first cycle, TsOH can protonate the labile Re–OH of the mono-condensed intermediate 8 to an aquo ion pair [8H]^0^·TsO^−^ (+14.9), with water then leaving. The diol ring can then close onto the low-coordinate rhenium centre without a barrier, delivering the diolate 10 after barrierless return of the proton to tosylate (Fig. 4). Dehydrating first removes the +42.1 kcal mol^−1^ condensation barrier TS8-9 of Fig. 2b. The same procedure also lowers the reduction barrier: TsOH protonates 11 and removes its water to give the neutral complex 11^DEHYD^ (water dissociation is barrierless, Section S5.2), and the β-hydride transfer proceeds through the neutral saddle TS11-7(TsOH) at +30.4 kcal mol^−1^, *versus* +63.2 for the bare anion. Protonation and water removal convert an anionic, electron-rich rhenium centre that cannot accept a hydride into a neutral one that can. The predicted mechanism therefore follows the condensation-first sequence already supported for the case of oxorhenium catalysts with sulfite and phosphine reductants;^6,10^ but, unlike previous modelling, the mechanism is consistent with experimental rates and suitably explains the role of acid.

**Fig. 4:**
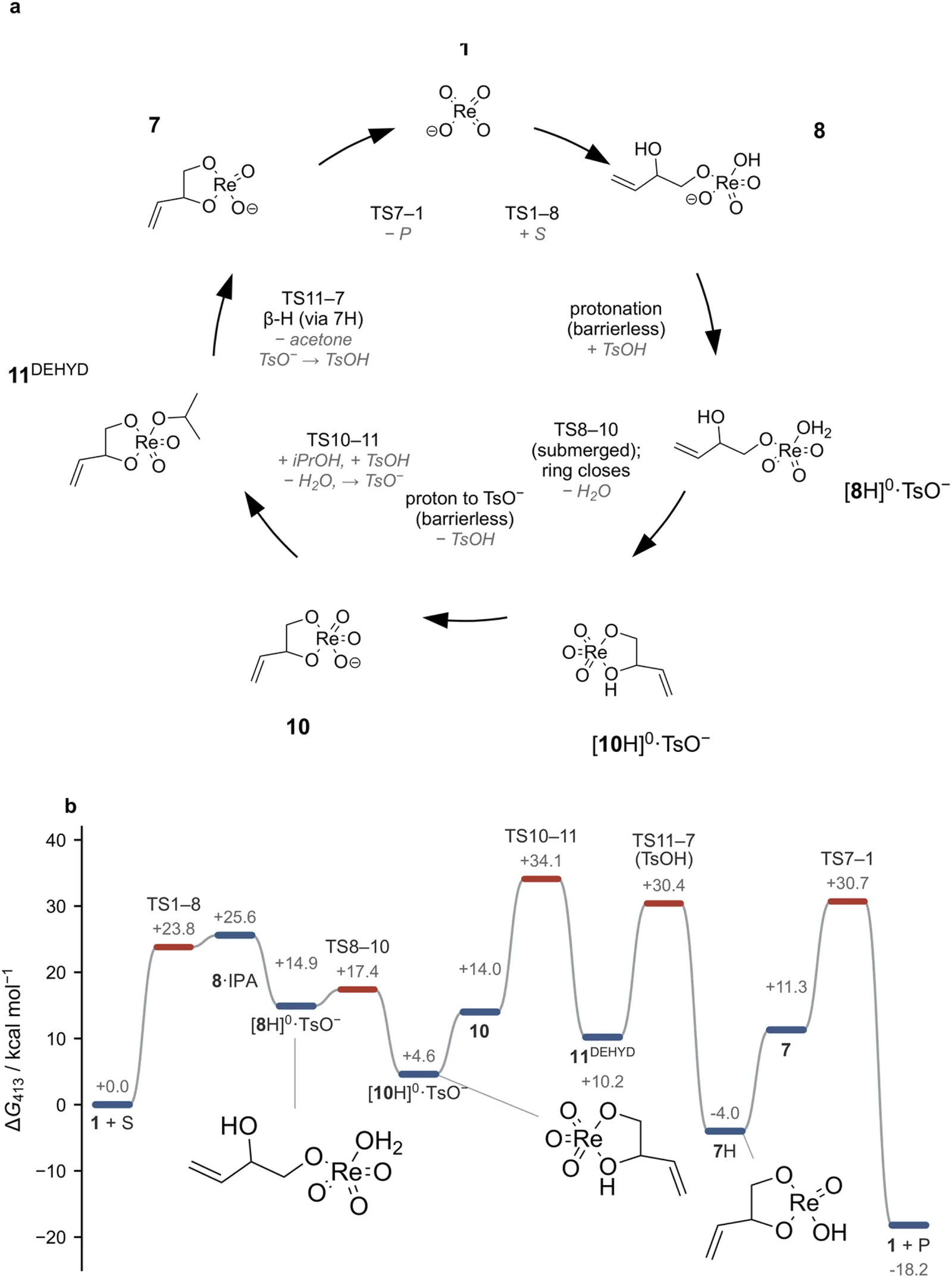
Acid-assisted mechanism. (a) Catalytic cycle. (b) Free energy profile. *S* is the diol substrate, and *P* is the alkene product.

Unlike the proton-free mechanisms, this route gives rates comparable with experiment. The full microkinetic model gives an apparent Δ*G*^‡^_eff_ of 30.3 kcal mol^−1^ at 413 K, a time to 50% conversion of ≈8 h at 3 mol% loading and a resting state that is 56% bare monomeric perrhenate, with the remainder split between the post-dehydration ion pair (24%) and the protonated product complex 7H (20%). Switching the acid off (removing the proton source rather than diluting it) collapses the rate to the acid-free floor of Section 3.1: the effective barrier Δ*G*^‡^_eff_ increases again to 45.8 kcal mol^−1^, a gap of 15.5 kcal mol^−1^, or 8.2 orders of magnitude for the rate at the operating temperature. Because the rate is first order in acid only at low loading (below about 3 mM, see Fig. S6), diluting the acid lowers the rate. This is particularly relevant to experiments that add no acid beyond the weak acid used as a counterion for perrhenate (often ammonium). See Section 3.3.

The control of the reaction rate on the operative pathway is distributed among three points: the site where the acid enters (the protonation of 8, *X*_RC_ = 0.43), the isopropanol-binding relay TS10-11 (0.38) and the cycloreversion TS7-1 (0.20), none of which accounts for more than half the control. Although the protonation of 8 has no electronic barrier and is treated at the diffusion limit (Section S6.2), it still exerts control over the rate because the encounter between TsOH (0.03 M) and 8, which is a minor species in the cycle, is one of the slowest events in an absolute sense. This is also the reason why the reaction is first order in acid when the acid loading is low (Section S6.4).

The acid facilitates the two steps that follow a loss of water from a Re–OH group, namely the ring closure at 8 and the β-hydride transfer at 11. In the absence of acid, the ring closure involving the transition state TS8-9 (with an energy of +42.1 kcal mol^−1^) involves a nucleophilic attack by the second diol oxygen on the five-coordinate rhenium atom, together with a proton shift from the nucleophilic O atom to an oxo ligand *via* isopropanol. Following protonation and the loss of water, the ring closure becomes a simple nucleophilic attack on the four-coordinate rhenium, forming a dative bond (as opposed to a covalent bond in the case of the acid free mechanism) and no coupled proton transfer. The acid-activated dehydration (previous step) created the possibility of this lower barrier step involving the formation of a dative bond with no proton transfer.

In the case of the reduction step, an activation-strain analysis^51^ indicates that the decrease in energy is due to a strain effect: the neutral rhenium centre becomes a better hydride acceptor, thus allowing for an earlier transition state (the C–H distance at the transition state is decreased from 1.45 Å to 1.36 Å) and therefore less destabilisation due to strain (see Section S5.5 and Fig. S4).

### 3.3 Kinetics compared to experimental data

Although there is no quantitative kinetic study available for the reaction between homogeneous perrhenate and an alcohol acting as a reductant, the apparent energy barriers can be estimated from the reported conditions and yields by using the inverse of the Eyring equation. For the ten perrhenate-catalysed reactions that have been described, four of these involving ammonium perrhenate and either benzyl alcohol or 3-octanol at 145–170 °C (ref. 7) and six involving sulfite together with three perrhenate salts at 150 °C,^6^ the values obtained are 31.5 to 34.0 kcal mol^−1^ (with a median of 33.0; Section S6.5 and Table S7) when the loading is taken as the number of active sites; if instead only one-tenth of the rhenium is assumed to take part in the catalytic cycle, then each of the inferred values rises by 1.9 kcal mol^−1^. Perrhenate-like ReO_*x*_ nanoparticles which convert glycerol in the presence of 3-octanol yield a measured value of 34.1 kcal mol^−1^ under the same 413 K convention,^52^ while MTO has a value of about 31 kcal mol^−1^, even though that system is neutral and dimeric.^12^

Fig. 5 summarises the results by comparing predicted rates and/or effective barriers from different mechanistic models with experimental values. The single-alcohol acid-free cycles have barriers that are never lower than +63 kcal mol^−1^; the second isopropanol lowers the barrier to 45.8 kcal mol^−1^; the proton lowers the modelled effective activation free energy to 30.3 kcal mol^−1^ (Table S6). Computed activation free energies can be compared to the approximate experimental operational window (built from 10 reported turnovers; Section S6.5). Because the reaction slows as its co-products build up (two equivalents of water and one of acetone per turnover; water inhibits the proton-gated steps with an order near −1, and acetone keeps rhenium as 7H; Section S6.4), we report the modelled activation barrier at 0.1%, 1% and 10% conversion, for comparison to experiment. Under these conditions, the modelled activation barrier increases from 30.3 to 31.5 kcal mol^−1^ at 1% conversion, and to 33.4 at 10% conversion, bracketing the window median of 33.0. At higher conversions, the cycloreversion TS7-1 becomes the leading carrier of rate control, ahead of the isopropanol-binding relay, as the acid-entry pre-equilibrium fills (Section S6.4). Integrated in time as a batch reactor (Fig. S5), the model shows that 7H builds up to about half of the rhenium as acetone accumulates, the rest being perrhenate 1 and, briefly at the start, 10H·TsO^−^.

**Fig. 5:**
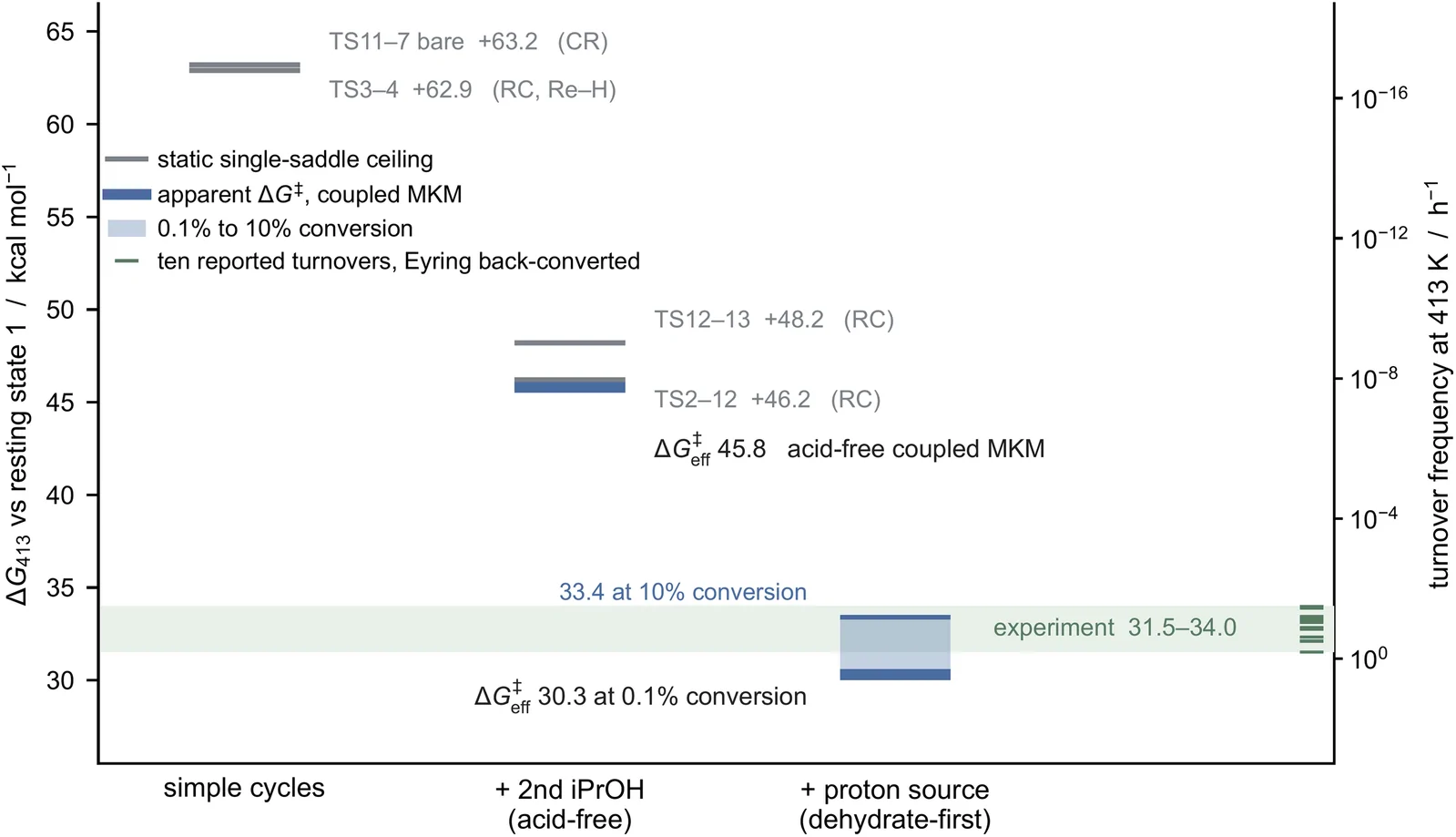
Summary of results. Grey levels are the highest saddle of each static profile; blue levels are the multi-step apparent Δ*G*^‡^_eff_ from the coupled microkinetic model. The 45.8 kcal mol^−1^ rung is the acid-free coupled network; the operative acid-enabled mechanism is drawn as a band spanning the conversion range over which the comparison with batch data is made: the solid lower edge is 30.3 at the 0.1% initial-rate probe, and the upper edge is 33.4 at 10% conversion, the apparent barrier rising with conversion because each turnover releases two equivalents of water and one of acetone. The green band is the 31.5–34.0 kcal mol^−1^ operational window; the ten Eyring back-converted turnover reports are marked as ticks; the right-hand axis converts Δ*G*^‡^ into turnover frequency at 413 K.

One computed barrier can be checked directly against experiment. Gable measured the extrusion step in isolation, on well-defined monomeric Re(v) diolates of Cp*Re(O) (313–323 K) and Tp′Re(O) (373–393 K), and found Δ*G*^‡^ = 24.5–29.1 kcal mol^−1^ with Δ*S*^‡^ ≈ 0. Hence, the barriers are effectively temperature-independent and comparable with ours at 413 K.^53^ On our converged surface the same step has a barrier of 19.4 kcal mol^−1^ from the diolate 7 to TS7-1 at 413 K, which looks 5 kcal mol^−1^ too low until the substrate is accounted for: our butenediolate gains 6.4 kcal mol^−1^ of allylic stabilisation in the extrusion transition state that Gable's saturated diolates cannot gain, and adding that term back puts the saturated analogue at 25.8, inside the measured range. While differences in ligands limit the precision expected from such a comparison, they provide additional evidence for the accuracy of the modelling.

We note that the overall reaction order for the MTO catalyst has been measured and is 1/2. Our predicted mechanism here would be first-order in perrhenate. This difference is accounted for by the fact that MTO can be shown to exist in the resting state as a dimer.^12^ Our calculations confirm that MTO can form a stable dimer, but for perrhenate, dimerisation is very endothermic at roughly +32 kcal mol^−1^ for electrostatic reasons (see Section S7).

## 4. Conclusions

We have described a mechanism for perrhenate DODH that is kinetically realistic. The operative cycle is condensation-first and acid-activated at its water-loss steps: a proton converts a labile Re–OH into a water molecule that departs, ahead of both diol ring closure and alcohol reduction, thereby converting an anionic centre that cannot effectively perform either step into a neutral one that does both easily. While tosylic acid shows the strongest effect, weaker acids also allow this mechanism (Section S5.4). On coupled-cluster-anchored energies, the microkinetic model lands inside the experimental window. No single step is rate-determining.

The predicted mechanism suggests that acid concentration should match rhenium loading, beyond which the rate saturates (Section S8.1 and Fig. S6), and that the reaction can be greatly accelerated by removing the water and ketone by-products (Section S8.2 and Fig. S7). The predicted mechanism also suggests that some heterogeneous DODH catalysts display “boomerang” behaviour (Section S8.3 and Fig. S8).

The second conclusion from this work is the usefulness of an agentic-AI workflow. An agentic mechanism search, in which the authors defined the candidate mechanisms and the agent carried out the transition-state searches and calculations, accelerated the mechanistic investigation, allowing us to explore many possible options for lowering the mechanism's activation energy to levels consistent with experimental data. All 44 transition states located for this project were located through the agentic-AI workflow, starting from routes supplied by the authors; the searches themselves needed no human help, and the authors validated each result.

Furthermore, this investigation has highlighted that the solvent must be resolved shell by shell, step by step, at the reaction temperature, rather than applied as a uniform correction.

Finally, we have highlighted that microkinetic modelling is indispensable for determining whether a given modelled pathway is realistic.

## Author contributions

J. M. S.: conceptualisation, data curation, formal analysis, investigation, methodology, software, validation, visualisation, writing – original draft, writing – review and editing. J. N. H.: conceptualisation, funding acquisition, project administration, resources, supervision, writing – review and editing.

## Conflicts of interest

There are no conflicts to declare.

## Supplementary Material

SC-OLF-D6SC06865K-s001

## Data Availability

Supplementary information (SI): contains the computational details and the agentic search protocol; the level-of-theory benchmarks and diagnostics; the conformational sampling; the four-*versus* six-centre proton-transfer and acid-leg analyses; the microkinetic model specification with its results and robustness tests; the dimerisation analysis; the reaction design lessons; and the supplementary figures. See DOI: https://doi.org/10.1039/d6sc06865k. Data for this article, including optimised geometries as xyz files, the species map, the full energy tables for every profile, the level-of-theory benchmark matrix, the electronic-structure analyses and the microkinetic model input and code, are available at Zenodo at https://doi.org/10.5281/zenodo.22031393. The agentic mechanism-search skill is available at Zenodo at https://doi.org/10.5281/zenodo.22030968. A maintained version is available at https://github.com/joshazerty/chem-skills.
