# Supplementary material for: Acid-assisted water loss in perrhenate-catalysed deoxydehydration: an agent-assisted reaction-network search with microkinetic modelling
Source: Chem Sci. 2026 Oct 27. Online ahead of print. doi: 10.1039/d6sc06865k (PMC13720772; doi:10.1039/d6sc06865k)
Supplement: Supplementary file 1 [file SC-OLF-D6SC06865K-s001.pdf]

Supplementary Information (SI) for Chemical Science.
This journal is © The Royal Society of Chemistry

Supplementary Information

Acid-assisted water loss in perrhenate-catalysed deoxydehydration: an agent-assisted reaction-network search with microkinetic modelling

Joshua M. Sims*[a] and Jeremy N. Harvey[a]

[a] Department of Chemistry, KU Leuven, Celestijnenlaan 200F, B-3001 Leuven, Belgium. E-mail: joshua.sims@kuleuven.be

All energies are reported in kcal mol^{-1} and, unless otherwise specified, correspond to quasi-harmonic Gibbs free energies at 413.15 K relative to bare perrhenate **1**.

Contents

- S1. Computational details and search protocol
- S2. Level of theory: benchmarks and diagnostics
- S3. Conformational sampling
- S4. Proton transfer: four- versus six-centre transition states
- S5. Acid chemistry
- S6. Microkinetic model
- S7. Dimerisation
- S8. Reaction design lessons
- S9. Supplementary figures
- S10. References

S1. Computational details and search protocol

Electronic structure. All geometries and frequencies were calculated using Gaussian 16 [1] at the B3LYP-D3(BJ)/def2-SVP level [2–4] for all atoms except rhenium. For rhenium, the Stuttgart–Dresden (SDD) relativistic effective core potential and its associated valence basis were employed [5]. Calculations were performed in PCM(1-pentanol) [6] with an UltraFine integration grid and quadratically convergent SCF. Unless otherwise specified, all species are closed-shell singlets with a net charge of −1, and minima exhibit no imaginary frequencies. Each transition state was validated by three criteria: the presence of exactly one imaginary frequency, a normal mode corresponding to the intended bond-forming or bond-breaking coordinate, and an intrinsic reaction coordinate [7] leading to the relevant species.

Free energies. Three corrections are applied to the rigid-rotor/harmonic-oscillator value: (i) quasi-harmonic vibrational entropy (GoodVibes [8]), in which all vibrational frequencies below 50 cm^{-1} are raised to 50 cm^{-1} [9]; (ii) the harmonic free energies are calculated referring to a 1 M solution (except isopropanol, for which the concentration is neat at 13.06 M); and (iii) tunnelling, which is treated as a Wigner correction [10] on each saddle, is now expressed in the analytic Skodje–Truhlar [11] form specifically for the two sharp β-hydride saddles. The energies are given at 413.15 K, which is the experimental operating temperature.

Agentic search. The search was conducted in iterative cycles. In each cycle, the hypotheses to be tested were identified by the authors and a target barrier was established, based on a synthesis of relevant literature and results from the preceding cycle. Hypotheses included the possibility of an alcohol relay for each proton transfer, protonation of Re–OH or Re=O by TsOH, involvement of alkali or ammonium counter-ions, use of alternative reductants such as 3-pentanol, and a dinuclear $Re_2O_7^{2-}$ pathway. Subsequently, the large-language-model agent (Claude Code) executed the following steps for each elementary step proposed by a hypothesis, following a fixed written protocol.

Endpoints. The reagents and/or the products of each step are given to the agent as multimodal input (in the form of a drawn reaction scheme, an image or a written description). Intermediates not described by the authors but deducible from the mechanistic route they have described are deduced by the agent. When needed, the agent optimised the endpoint geometries of the relevant step and carried out a conformer search using CREST (see Section S3). The two endpoint geometry files are built with identical atom ordering (a prerequisite for the growing string method implemented for locating the transition state).

Transition state. A growing-string calculation [12] with g-xTB (11 nodes) provides an initial transition-state guess. If the string fails, the endpoints are modified, and NEB-TS in ORCA is employed instead. The guess is then optimised as a transition state, including frequency calculations, at the production level (B3LYP-D3(BJ)/def2-SVP/SDD, PCM(1-pentanol)). As a fallback option, the growing string calculation is performed again at the DFT level of theory.

Acceptance. A transition state is accepted if it meets the following criteria:

(i) It has only one imaginary frequency.

(ii) The vibrational mode corresponding to this imaginary frequency matches the expected bond changes for the step being studied.

(iii) IRC calculations in both directions give rise to chemically different endpoints, which, after re-optimisation, correspond to the intended reactant and product.

All the structures that were retained have been checked by the authors.

Scope. Approximately 1,500 frequency calculations were performed. A total of 111 stationary points were retained for both diols, including 44 validated transition states. Main-text Scheme 1 provides a summary of the workflow.

S2. Level of theory: benchmarks and diagnostics

Composite protocol. Converged energies substitute higher-level electronic energies into the production quasi-harmonic thermochemistry, $G = E(\text{level}) + [G_{qh}(\text{B3LYP}) - E(\text{B3LYP})]$, on the B3LYP geometries. Two levels are used: PBE0-D3(BJ)/def2-TZVP (PCM and gas-phase single points, Gaussian throughout) [13] and DLPNO-CCSD(T) [14] extrapolated to the basis-set limit [15] (def2-TZVPP→def2-QZVPP, TightPNO; ORCA [16]), applied as anchors (CC differentials) on fourteen saddles: **TS2-12**, **TS2-14**, **TS3-4**, **TS7-1**, **TS8-9**, **TS8-10**, **TS9-10**, **TS10-11**, **TS11-7** (bare), **TS11-7** (TsOH), **TS12-13**, **TS14-4**, **TS1-8** and **TS4-5**. Eight are folded into the microkinetic networks as CC–PBE0 differentials: **TS2-12** (+1.25), **TS12-13** (+5.07), **TS7-1** (+2.48) on the reduction-first network, and **TS8-9** (−0.99), **TS9-10** (+4.86), **TS10-11** (+1.60), **TS11-7** (TsOH) (+4.73) on the condensation-first network, with **TS8-10** (+0.1) on the dehydrate-first leg. Four anchor the static profiles only, namely **TS3-4** (+2.83), **TS2-14** (+6.25) and **TS14-4** (−0.51), because those saddles lie on the oxo/hydride redox-isomer branches, which are gated by entries roughly 30 kcal mol^{-1} above the operative ceilings (**TS3-4** +62.9 and **TS2-14** +66.8 on the corrected standard state, against ceilings of +30–34) and are therefore not carried as nodes in the kinetic networks; **TS14-4** (+41.2) lies downstream of that inaccessible gate on the same branch; the same applies to **TS11-7** (bare), whose ion-paired analogue **TS11-7** (TsOH) is the operative node. For **TS1-8** and **TS4-5**, the anchor is reported, but the differential is deliberately not folded into the network: both carry a degree of rate control of exactly zero in every scenario of Section S6, so the differential cannot alter any computed observable, and for **TS4-5** the coupled-cluster value is in any case treated as authoritative in preference to PBE0 (PBE0's −4.80 kcal mol^{-1} gas-phase error on this step is the largest in this work, and node **4** carries an elevated T1). The geometry channel was checked by re-optimising **TS2-12** at PBE0: reactant and saddle relax in parallel, and the barrier moves by ≈ −0.4 kcal mol^{-1}, so single points on B3LYP geometries are adequate.

Gaussian–ORCA switching. Gaussian-PCM and ORCA-CPCM absolute energies are not interchangeable for these rhenium anions, with discrepancies reaching up to 28 kcal mol^{-1} for a single absolute point (as observed for intermediate **10**, which was evaluated by both methods). Consequently, each composite step employs the same-code PCM and gas-phase pairs, ensuring that the offset largely cancels within the same-code step difference.

Basis-set superposition error. Counterpoise analysis of the associative condensation step indicates that def2-SVP over-binds by +8.0 kcal mol^{-1} per isopropanol contact, compared to +1.7 kcal mol^{-1} with def2-TZVP. The split-valence basis thus excessively stabilises certain

structures, particularly those with multiple hydrogen bonds, such as six-membered-ring transition states. To address this, the triple-ζ basis was used to achieve convergence; transitioning from TZVP to QZVP results in a shift of ≤ 0.3 kcal mol⁻¹.

Coupled-cluster validation of condensation saddles. The PBE0/def2-TZVP calculations reproduce the condensation barriers obtained with DLPNO-CCSD(T)/CBS within approximately 1 kcal mol⁻¹, confirming that these saddles are single-reference in character.

Table S1. Coupled-cluster validation of the condensation saddles: PBE0/def2-TZVP local barriers against DLPNO-CCSD(T)/CBS, with the PBE0 error and the T1 diagnostic (gas phase, B3LYP geometries). Energies in kcal mol⁻¹.

step (gas, local)	PBE0/def2-TZVP	DLPNO-CCSD(T)/CBS	PBE0 error	T1
**TS2-12** (RC 2nd condensation)	+10.85	+12.10	−1.25	0.0175
**TS8-9** (CR 2nd condensation)	+17.13	+16.14	+0.99	0.0186
**TS1-8** (CR 1st condensation)	∼+0.6	+1.62	∼−1	0.0185
**TS4-5** (RC 1st condensation)	−3.28	+1.52	−4.80	0.0193 (node **4**: 0.0277)

The cycloreversion is functional-sensitive. Thirteen functionals [17] were benchmarked on the retro-[3+2] local barrier (**TS7-1** – **7**, def2-TZVP, gas, on the production B3LYP geometries) against a DLPNO-CCSD(T)/CBS reference. They span 27.7 kcal mol⁻¹, from +1.2 to +28.9, and the coupled-cluster value of +17.2 sits near the centre of that range rather than at either edge:

*Table S2 Thirteen-functional benchmark of the retro-[3+2] cycloreversion barrier (**TS7-1** – **7**; def2-TZVP, gas phase, B3LYP geometries), ordered by barrier height and referenced to DLPNO-CCSD(T)/CBS. Energies in kcal mol⁻¹.*

method (def2-TZVP)	barrier	dev. vs CC
M06-2X	+28.9	+11.7
ωB97X-V	+28.5	+11.3
ωB97X-D3(BJ)	+28.1	+10.9
ωB97M-V	+27.4	+10.2
M06	+18.7	+1.5
**DLPNO-CCSD(T)/CBS**	**+17.2**	–
PW6B95-D3(BJ)	+16.0	−1.2
PBE0-D3(BJ)	+14.7	−2.5
B3LYP-D3(BJ)	+9.2	−8.0
revDSD-PBEP86-D4	+6.5	−10.7
M06-L	+4.7	−12.5
TPSSh-D3(BJ)	+4.2	−13.0
B2PLYP-D3(BJ)	+1.5	−15.7
r2SCAN-D4	+1.2	−16.0

Four functionals collapse the step, falling 12.5–16.0 kcal mol⁻¹ below coupled cluster (r2SCAN-D4, B2PLYP, TPSSh, M06-L); the range-separated hybrids and M06-2X overshoot it by 10.2–11.7; only PW6B95, M06 and PBE0 come within 2.5. The error is specific to this step: the same thirteen functionals span only 3.7 kcal mol⁻¹ for hydride elimination and 7.2 kcal mol⁻¹ for CR β-hydride transfer.

Origin of the functional spread. The cycloreversion barrier was recalculated using a PBE-based hybrid functional, varying the fraction of exact exchange (a_x) from 0 to 0.60 (def2-TZVP, gas phase, ORCA). The barrier increases linearly with a_x, rising by 78.5 kcal mol⁻¹ per unit increase (R^2 = 0.9997), from −4.1 at a_x = 0 to +42.9 at a_x = 0.60 (see Figure S2a). The global hybrids and meta-GGAs listed in Table S2 align with this trend according to their exact exchange fractions (r2SCAN and M06-L at 0, TPSSh at 0.10, B3LYP at 0.20, PBE0 at 0.25, M06 at 0.27, PW6B95 at 0.28, M06-2X at 0.54). Range-separated hybrids, which reach full exact exchange at long range, are 10 to 11 kcal mol⁻¹ higher than the coupled-cluster value, while the two double hybrids are 11 to 16 kcal mol⁻¹ lower. The same thirteen functionals show much smaller spreads for other coupled-cluster-anchored barriers in the benchmark set (Figure S2b): 3.7 (**TS3-4** – **3**), 7.2 (**TS11-7**(TsOH) – **11**$^{\mathbf{DEHYD}}$), 10.0 (**TS12-13** – **12**), and 10.0 kcal mol⁻¹ (**TS11′-7′** – **11′**). For the five global functionals (r2SCAN, TPSSh, B3LYP, PBE0, and PW6B95) the slope with respect to a_x is +55 kcal mol⁻¹ per unit for **TS7-1** and between −5 and +10 for the other barriers. This dependence on exact exchange is characteristic of delocalisation error [18] rather than static correlation, which is examined below. One possible explanation for the concentration of this effect at this step is that cycloreversion involves a change in the formal oxidation state of rhenium (from Re(V) to Re(VII) as the alkene is released).

Wavefunction diagnostics. Single-reference character was tested for the cycloreversion saddles **TS7-1** and **TS7′-1** (propane-1,2-diol), together with their reactants **7** and **7′**, the Re(V) species **3** and **4**, the saddles **TS3-4**, **TS12-13** and **TS11-7**(TsOH), and closed-shell ReO_4^- (**1**) as a reference. The T1 diagnostics [19] derived from the DLPNO-CCSD(T)/def2-TZVPP calculations vary between 0.017 and 0.030 for all the rhenium species in the benchmark set, including 0.025 for ReO_4^-, as compared to 0.009–0.010 for the organic species. These values are the same for all the rhenium species and are below the threshold of 0.05 which has been proposed for transition-metal compounds [20] (Table S3). Furthermore, a CASSCF(10,10) calculation located the triplet state of **TS7-1** 37.6 kcal mol⁻¹ above the singlet state and 24.0 kcal mol⁻¹ above it at **7**. The singlet–triplet gap thus increases from the reactant to the saddle, and there is no requirement to consider any crossing between the spin surfaces along the cycloreversion. At the composite single-point level (PBE0-D3(BJ)/def2-TZVP(SDD)/PCM, Gaussian 16), the restricted closed-shell wavefunctions of **1**, **4**, **7**, **TS7-1**, **7′** and **TS7′-1** are stable with respect to spin-symmetry breaking, and broken-symmetry initial guesses on **7**, **TS7-1**, **7′** and **TS7′-1** converge back to the closed-shell solution. Overall, these tests show that there is no significant multireference character at **TS7-1** and the DLPNO-CCSD(T)/CBS barrier of +17.2 kcal mol⁻¹ is therefore retained as the reference.

Table S3. *DLPNO-CCSD(T)/def2-TZVPP T1 values.*

species	T1
**1** (ReO_4^-)	0.0253
3	0.0223
**TS3-4**	0.0224
**4** (ReO_3^-)	0.0277
7	0.0217
**TS7-1**	0.0278
7′	0.0220
**TS7′-1**	0.0295
**TS12-13**	0.0212
**TS11-7**(TsOH)	0.0244
iPrOH	0.0090
but-3-ene-1,2-diol	0.0104

S3. Conformational sampling

Conformational sampling was performed by generating ensembles using CREST metadynamics [21] with the GFN2-xTB/ALPB [22] model. Ensemble members within a 6 kcal mol⁻¹ xTB energy window were re-optimised at the production level, initially by freezing the reacting core, followed by full saddle optimisation of the remaining structures. These were re-ranked using composite criteria based on PBE0-D3(BJ)/def2-TZVP single-point calculations. Validation included confirming the correct number of imaginary frequencies

(NImag), visually inspecting the imaginary mode against the reference, and performing IRC verification for any saddle conformer considered for adoption.

S4. Proton transfer: four- versus six-centre transition states

In the main catalytic cycles, each proton transfer was observed both as a direct four-centre transfer and as a six-centre transfer relayed through isopropanol. In the latter, a spectator alcohol molecule accepts the proton at one end and donates it at the other.

Footing. The relay gap is defined as Δ = *G*(6m) − [*G*(4m) + *G*(iPrOH)], where the 4m reference releases exactly one isopropanol; composition was verified to be exact for all eight pairs (6m − 4m = C_3H_8O). Because the two sides differ by one free alcohol, the gap is **linear in the standard state chosen for free isopropanol, and that term does not cancel**. Isopropanol is the solvent, not a dilute reagent, so the neat-alcohol reference of Section S1 applies. All gaps below carry that term. The gaps are further evaluated on the composite footing declared in Section S2 (PBE0-D3(BJ)/def2-TZVP single points on the B3LYP geometries) rather than at the def2-SVP level at which the pairs were first located; def2-SVP over-binds the hydrogen-bonded relay ring, and since the 6m form carries one more alcohol contact than its 4m twin, it collects that error while the twin does not. The measured basis-set contribution is +7.05 kcal mol^{-1} on average (range +5.33 to +8.75), consistent with the +8.0 → +1.7 per-contact estimate of Section S2.

Temperature dependence. The relay is an enthalpy-for-entropy trade and is therefore temperature-dependent. Quasi-harmonic free energies were recomputed for all seventeen species at eight temperatures from 298.15 to 503.15 K (GoodVibes, same settings as Section S1). Every gap is linear in *T* over that range (R^2 ≥ 0.9999), giving Δ*H* and Δ*S* directly:

Table S4 Temperature dependence of the four- versus six-centre relay gap Δ for the eight proton-transfer pairs: ΔH, ΔS, the slope dΔ/dT, the gap at 413 K, and the crossover temperature T(Δ = 0).

pair	Δ*H* / kcal mol^{-1}	Δ*S* / cal mol^{-1} K^{-1}	dΔ/d*T* / kcal mol^{-1} per 100 K	Δ(413 K)	*T*(Δ=0) / K
**TS10-11**	−28.58	−29.68	+2.97	−16.30	963*
**TS10′-11′**	−19.36	−28.23	+2.82	−7.67	686*
**TS5-6**	−17.08	−28.30	+2.83	−5.36	603*
**TS6-7**	−14.53	−27.31	+2.73	−3.23	532*
**TS9-10**	−14.37	−29.50	+2.95	−2.17	487
**TS4-5**	−10.44	−26.38	+2.64	+0.48	396
**TS1-2**	−10.87	−28.21	+2.82	+0.81	385
**TS1-8′**	−9.84	−26.39	+2.64	+1.09	373

Asterisked crossover temperatures are linear extrapolations beyond the sampled range and are indicative only.

The eight rings share two key features. First, the entropic penalty is **nearly constant** among them, ranging from −26 to −30 cal mol^{-1} K^{-1}, reflecting the typical cost of incorporating a solvent molecule from the bulk into a bound transition state. Consequently, the relay benefit decreases at a similar rate for **each pair, and the temperature-dependent preference is primarily determined by Δ*H*.** Second, the enthalpic advantage varies widely (from −9.8 to −28.6 kcal mol^{-1}), resulting in crossover temperatures from 373 K to well beyond the experimentally accessible range. Four of the eight crossover temperatures fall between 373 and 487 K, encompassing the 363–443 K interval relevant to this chemistry. At the modelled temperature of 413.15 K, three relays retain an advantage of at least 5.4 kcal mol^{-1}, **TS6-7** has a smaller advantage of 3.2 kcal mol^{-1}, and four are within ±2.2 kcal mol^{-1} of their four-centre counterparts, making them indistinguishable by this method. We note that due to the use of a continuum model, which mixes enthalpic and entropic contributions to solvation yet appears purely in the form of an “enthalpic” term in the obtained results, the above separation of enthalpic and entropic terms is not rigorous, but it does usefully highlight the fact that incorporating an extra solvent molecule has a large and negative impact on entropy.

The way in which the relay saddles are positioned on the profiles for different species is subtly determined. The various ones (**TS5-6**, **TS6-7**, **TS9-10**) are at or below an adjacent intermediate when compared with the separated reactants together with free isopropanol,

since the neat-alcohol standard-state term causes these reference points to be raised. On the supermolecule (alcohol-bound) footing, they move away from their neighbouring wells. With respect to its association well, **TS6-7** represents a true barrier of +14.7 kcal mol^{-1} and **TS5-6** a small one of +1.8 kcal mol^{-1}, whereas **TS9-10** is situated even below its bound well. All of the submerged saddles are shown at their calculated values on the main profiles; none of them has a rate-controlling effect (Section S6.4).

S5. Acid chemistry

S5.1. The dead end: proton relay onto the condensation. On the gating CR condensation saddle **TS8-9**, explicit TsOH transfers its proton to the condensing alkoxide and the system relaxes to a stable ion pair (binding −9.3 kcal mol^{-1}, PBE0/def2-TZVP/PCM); the located stationary point is a minimum, and a scan of the putative proton→oxo advance climbs monotonically. The acid grips the substrate rather than advancing it.

S5.2. The second dehydration is barrierless. The water loss which precedes the reduction (**11** → **11**^{DEHYD}) has no identifiable kinetic saddle point due to its endergonic nature (+6.8 kcal mol^{-1}). When a relaxed scan is carried out along the Re–OH$_2$ coordinate, it rises continuously towards the dissociation asymptote.

S5.3. Outer-shell microsolvation control. The two shells act on the barriers in opposite directions. Two explicit outer-shell solvent molecules are fully relaxed on the gating condensation saddles and on their reactants, the barriers are shifted by +7 kcal mol^{-1} (**TS8-9**) and by approximately +1 (**TS2-12**); since the condensation transition states have fewer free hydrogen-bond donors than their reactants, the outer-shell solvent raises rather than lowers the barriers, whereas the participation of the first-shell relay in the proton transfers results in a lowering of the barriers by up to 16.3 kcal mol^{-1} at 413 K.

S5.4. The acid-activated pathway can likewise operate using weak acids; in the dehydration step, TsOH was replaced by a weak proton donor, namely the ammonium counterion of perrhenate, and the result is very similar to that obtained with TsOH, since weak acids are also capable of protonating node **8**.

S5.5. Origin of the acid effect at the ring-closure and reduction steps.

Ring closure. Without acid, the ring closes through **TS8-9** (+42.1 kcal mol^{-1}). In that instance, the step is a nucleophilic attack of the second diol oxygen onto the five-coordinated Re atom, and a simultaneous transfer of a proton, via isopropanol, to an oxo-ligand. In the acid-assisted mechanism, the nearest analogous step (**TS8-10**) is a nucleophilic attack without a simultaneous proton transfer, which requires much less in the way of bonding change. In more detail, the lowering of the barrier in the presence of acid can be explained as follows:

(i) In the acid-free mechanism, the step through **TS8-9** effectively requires an energetically unfavourable proton transfer from the diol O atom to one of the oxo ligands prior to any favourable ring formation, as shown by the fact that the bonding changes associated with proton transfer occur almost entirely before reaching the saddle point. At the saddle point, the Wiberg index [23,24] of the diol O–H bond has decreased to 0.07; that of the new O–H on the former oxo group is already as high as 0.69, whereas the new Re–O bond is still forming, with a Wiberg index of 0.54, compared to 0.70 in the product.

(ii) In contrast, in the acid-assisted mechanism where there is no proton transfer involved in **TS8-10** (which in fact starts with species **8H**) and the Re–O bond being formed is dative until the proton is removed in a further step, there is little change in chemical bonding during the step (the Wiberg index for the forming Re–O bond is only 0.16 at the saddle point, before further increases to 0.30 at **10H** and to 0.66 in species **10** after the proton has been removed).

(iii) Separating the proton transfer (and introducing a new base, TsO$^-$) elements from the Re–O covalent bonding changes explains why the activation barrier is lower in the presence of acid: the only changes to Re–O bonding incurred by **TS8-10** relate to dative interactions (coordination of an alcohol O; partial de-coordination of a departing water molecule) which presumably have lower intrinsic barriers.

Reduction. DODH involves a key metal reduction step which revolves around hydride transfer from the sacrificial alcohol reductant to an oxo ligand at the metal. The corresponding reduction barrier **TS11-7** is 12.0 kcal mol^{-1} lower in the case of the acid-assisted mechanism. This is because neutral **11**^{DEHYD} (from the acid-assisted mechanism) is a better hydride acceptor than anionic **11** (in the acid-free mechanism). This can be seen e.g. from the NPA/NBO charges [23] on Re, which are +1.76 in **11**^{DEHYD}, compared to +1.51

in **11**. The more facile hydride transfer is evident from the fact that the transition state is significantly earlier (the C–H distance is 1.36 Å at the TS in the case of the step starting from **11**^{DEHYD}, compared to 1.45 Å in the case of the step starting from **11**). The fragments therefore need less distortion to reach the TS. We have analysed this in terms of an activation strain model [25], and this shows that there is indeed a much lower degree of strain in the TS starting from **11**^{DEHYD} compared to that starting from **11** (−11.0 kcal mol^{-1}). This is partly offset by a lower gain in the interaction term (by 4 kcal mol^{-1}), but the dominant effect explains the higher reactivity (see Figure S4).

S6. Microkinetic model

S6.1. Specification and reproducibility. The reduction-first (RC) and condensation-first (CR) pathways are not treated separately; instead, they share the resting state **1**, the diolate **7** and the common product-extrusion saddle **TS7-1**, and therefore compete for the same amount of rhenium; as a result, they are considered as part of a single coupled network and are solved at quasi-steady state, with each intermediate maintained at its steady coverage and the resulting linear system being solved exactly rather than being integrated over time. The free species (isopropanol, water, acetone, TsOH and the diol) appear in the rate expressions as explicit activities on the standard-state chain described in Section S1, so that the neat-alcohol reference is consistently applied at every step. Each of the observable quantities is obtained at 0.1% conversion, which is near the initial rate. The concentration–time profiles (Figure S5) were calculated by integrating the same network, with the same rate constants and activity conventions, as for a closed batch reactor, starting from the reference initial conditions (Table S5: at t = 0 there is no water, acetone or butadiene and all the rhenium is in the form of **1**), with a BDF integrator (relative tolerance 10^{-8}, absolute tolerance 10^{-14} M), and rhenium is conserved to an accuracy of 10^{-11} M.

Table S5 Specification of the microkinetic model: initial conditions, reference activities, and the conventions adopted for fast steps, rate constants and the degree of rate control.

quantity	value	note
T	413.15 K	operating temperature
$[Re]_{total}$	0.03 M	3 mol% vs substrate
$[diol]_0$	1.0 M	but-3-ene-1,2-diol
[iPrOH]	13.0 M	neat reductant/solvent; reference activity 13.0
$[TsOH]_{total}$	0.03 M	equal to [Re] unless swept (S6.4)
[water], [acetone], [diene]	stoichiometric at the probe: 2x, x, x with x = 10^{-3} M	plus added water where swept
reference activities	iPrOH 13.0 M; all other solutes 1 M	the standard-state chain of Section S1
fast steps	diffusion-limited: ΔG‡ = 4.0 kcal mol^{-1}, k = 6.6×10^{10} s^{-1} at 413 K	barrierless proton transfers and conformational relaxations
rate constants	TST with Wigner tunnelling (Skodje–Truhlar on the β-hydride saddles)	Section S1
degree of rate control	Campbell [26], evaluated over every step, not only labelled saddles, by central finite difference (±0.5 kcal mol^{-1} on the transition-state free energy) at the quasi-steady state.	

S6.2. Rates of barrierless steps. The rates depend on how the barrierless steps are treated, specifically the diffusion limit in 1-pentanol at 413 K. It has been shown that diffusion limitation is roughly equivalent to a 4 kcal mol^{-1} barrier. This value was adopted throughout [27,28].

S6.3. Apparent Δ$G^{\ddagger}_{eff}$ at 413 K.

Table S6. Apparent free-energy barriers Δ$G^{\ddagger}_{\mathrm{eff}}$ at 413 K for each candidate surface, compared with the experimental window of 31.5–34.0 kcal mol⁻¹ derived in Section S6.5.

surface / model	Δ$G^{\ddagger}_{\mathrm{eff}}$	verdict vs window (31.5–34.0, Section S6.5)
B3LYP/def2-SVP baseline (coupled, acid at reduction)	22.4	too fast by ∼5 orders vs the window median (due to BSSE, Section S2)
single-alcohol acid-free cycles, highest static barriers	62.9 (**TS3-4**) / 63.2 (**TS11-7** bare)	not viable
best acid-free coupled network (TsOH = 0), **TS2-12** as published	45.8	too slow by ∼7 orders; rate control concentrated on the second condensation **TS2-12** (0.98)
operative: acid-enabled dehydrate-first	30.33	inside the window at matched conversion (Section S6.4)

S6.4. The operative route in detail (CC, 413 K, 0.03 M TsOH, probed at 0.1% conversion). Δ$G^{\ddagger}_{\mathrm{eff}}$ = 30.33 kcal mol⁻¹; TOF = 7.8 × 10⁻⁴ s⁻¹. Resting state: **1** 56%, **10H**·TsO⁻ 24%, **7H** 20%. Rate control is split between the acid protonation entry (X = 0.43), the isopropanol-binding relay **TS10-11** (0.38) and the cycloreversion **TS7-1** (0.20); no single step approaches unit control. Fitted initial-rate orders: [Re] 1.00 (exact), [iPrOH] 0.77, [TsOH] 0.55, [diol] 0.22. Acid dependence: the model is cleanly first order in TsOH below ∼3 mM (local order 0.96–1.04, holding down to trace concentrations, where the apparent barrier reads 31.8 at 3 mM, 33.6 at 0.3 mM and 37.4 at 3 μM), passes 0.55 at the 0.03 M reference loading, and saturates through 0.30 (0.1 M) to 0.12 (0.3 M); half-saturation falls near 0.03–0.05 M TsOH at this rhenium loading (Figure S6; see also Section S8.1). Water dependence: added water inhibits with an order of −1.0 (0.5–1 M window); Δ$G^{\ddagger}_{\mathrm{eff}}$ rises from 32.0 to 34.9 kcal mol⁻¹ across 0.1–4 M added water. Conversion dependence: the same effect makes the apparent barrier a function of conversion, because the reaction generates two equivalents of water per turnover. Probed at 0.1%, 1%, 10% and 50% conversion, the model reads 30.33, 31.5, 33.4 and 34.1 kcal mol⁻¹, and the rate control migrates off the acid-entry pre-equilibrium onto the cycloreversion **TS7-1** (0.51 at 1%, 0.64 at 10%), which becomes the leading carrier, and onto **TS10-11** (0.40 and 0.49), as the reservoirs fill and **7H** takes up 49–55% of the resting state (Figure S5).

S6.5. Derivation of the operational window. In order to make use of the published experimental data [29,30], the times and yields reported were converted into apparent free-energy barriers using transition-state theory, according to the equation $k_{\mathrm{app}} = (k_{\mathrm{B}} T/h)$ exp(−Δ$G^{\ddagger}_{\mathrm{app}}/RT$):

Table S7. Published deoxydehydration data converted to apparent free-energy barriers through transition-state theory, ordered by Δ$G^{\ddagger}_{\mathrm{app}}$, giving the 31.5–34.0 kcal mol⁻¹ operational window.

source	entry	catalyst	reductant	substrate	T / °C	t / h	basis	cat. / mol%	TOF / h⁻¹	Δ$G^{\ddagger}_{\mathrm{app}}$
Boucher-Jacobs 2013	T1 e10	NH_4ReO_4	3-octanol (neat)	glycerol	170	2.5	59% yield	2.5	9.44	31.5
Ahmad 2011	T1 e3	NH_4ReO_4	Na_2SO_3	1-phenyl-1,2-ethanediol	150	12	100% conv.	10	0.83	32.1
Boucher-Jacobs 2013	T1 e5	NH_4ReO_4	benzyl alcohol	diethyl tartrate	145	24	95% yield	10	0.40	32.3
Boucher-Jacobs 2013	T1 e3	NH_4ReO_4	benzyl alcohol	glyceryl ether	150	24	83% yield	10	0.35	32.8

source	entry	catalyst	reductant	substrate	T / °C	t / h	basis	cat. / mol%	TOF / h^{-1}	$\Delta G^{\ddagger}_{app}$
Ahmad 2011	T1 e15	NH_4ReO_4	Na_2SO_3	1,2-octanediol	150	26	100% conv.	10	0.38	32.8
Ahmad 2011	T1 e2	$NaReO_4$	Na_2SO_3	1-phenyl-1,2-ethanediol	150	40	100% conv.	10	0.25	33.1
Boucher-Jacobs 2013	T1 e1	NH_4ReO_4	benzyl alcohol	1,2-octanediol	150	24	50% yield	10	0.21	33.3
Ahmad 2011	T1 e4	Bu_4NReO_4	Na_2SO_3	1-phenyl-1,2-ethanediol	150	59	100% conv.	10	0.17	33.4
Ahmad 2011	T1 e16	Bu_4NReO_4	Na_2SO_3	1,2-octanediol	150	100	100% conv.	10	0.10	33.9
Ahmad 2011	T1 e20	Bu_4NReO_4	Na_2SO_3	1,2-tetradecanediol	150	110	100% conv.	10	0.09	34.0

Range 31.5–34.0, median 33.0, which is the window quoted in the main text. Six of the ten entries carry a stoichiometric protic cation (NH_4^+), which has been shown to follow the acid-activated route described here. The four aprotic-cation entries (one $NaReO_4$, three Bu_4NReO_4) all use the sulfite reductant and are reported for comparison.

S7. Dimerisation

The half-order rhenium dependence and dimeric resting state observed for MTO do not apply to perrhenate, due to the charge associated with the assembled dimer. Formation of the μ-oxo bis(diolate) dimer from the catalyst resting state incurs an energy cost of +31.8 kcal mol^{-1} for perrhenate, whereas it releases 20.1 kcal mol^{-1} for MTO. Thus, the dimer is a thermodynamically favourable resting state for MTO, consistent with its half-order rhenium dependence and the NMR evidence for a dinuclear resting state [31], while for perrhenate, the dimer lies significantly above monomeric ReO_4^-. Dimerisation is therefore prohibited for perrhenate for electrostatic reasons.

S8. Reaction design lessons

S8.1. Acid concentration. The predicted mechanism entails that the increase in turnover frequency as the acid concentration rises will saturate when the acid concentration is approximately equal to the rhenium loading (see Figure S6a). Furthermore, the apparent acid order is nearly identical to the fraction of Re present in the system in the form of ReO_4^- (see Figure S6b).

This means that, while the predicted mechanism has no optimal acid concentration, acid concentrations much greater than Re loading have little effect on reaction kinetics. In practice, this would have a negative effect as other reactions start to occur (for example, acid-catalysed ketalisation of the diol by the acetone co-product).

S8.2. Remove water and ketone. The predicted mechanism indicates that reaction kinetics will be enhanced by removal of water and ketone products. In a closed system, the effective barrier rises with conversion (30.3 kcal mol^{-1} at 0.1% conversion, up to 34.1 kcal mol^{-1} at 50% conversion). This is illustrated by the increase in the effective barrier when small quantities of water or acetone are added (see Figure S7a). If both products were eliminated as they form, the effective barrier would remain 30.2 kcal mol^{-1}. More specifically, the ketone inhibits the reaction by holding Re longer at **7H** (see Figure S7b), while water returns Re to **1** by reversing the proton-assisted condensation.

S8.3. Supported molecular catalyst boomerang effect. One possibility for performing heterogeneous DODH is to have a supported cation, which would then lead to ReO_4^- being electrostatically bound at the interface [32], a strategy also implemented for other oxoanion catalysts [33,34]. The predicted mechanism indicates that the system would operate via a “boomerang” effect due to charge changes throughout the catalytic cycle (see Figure S8). Protonation would allow the Re catalyst to leach into solution and be replaced at the interface by the tosylate anion. Further along in the catalytic cycle, when the Re anion is regenerated, it would return to the interface. Properly understanding that such catalysts exemplify “boomerang” behaviour is important, both for understanding catalytic activity (the catalyst cannot be properly understood or modelled as a solely homogeneous or heterogeneous catalyst) and also for understanding deactivation through leaching (this can occur not only by the cation+anion leaching into solution, but also by anion exchange at the interface).

S9. Supplementary figures

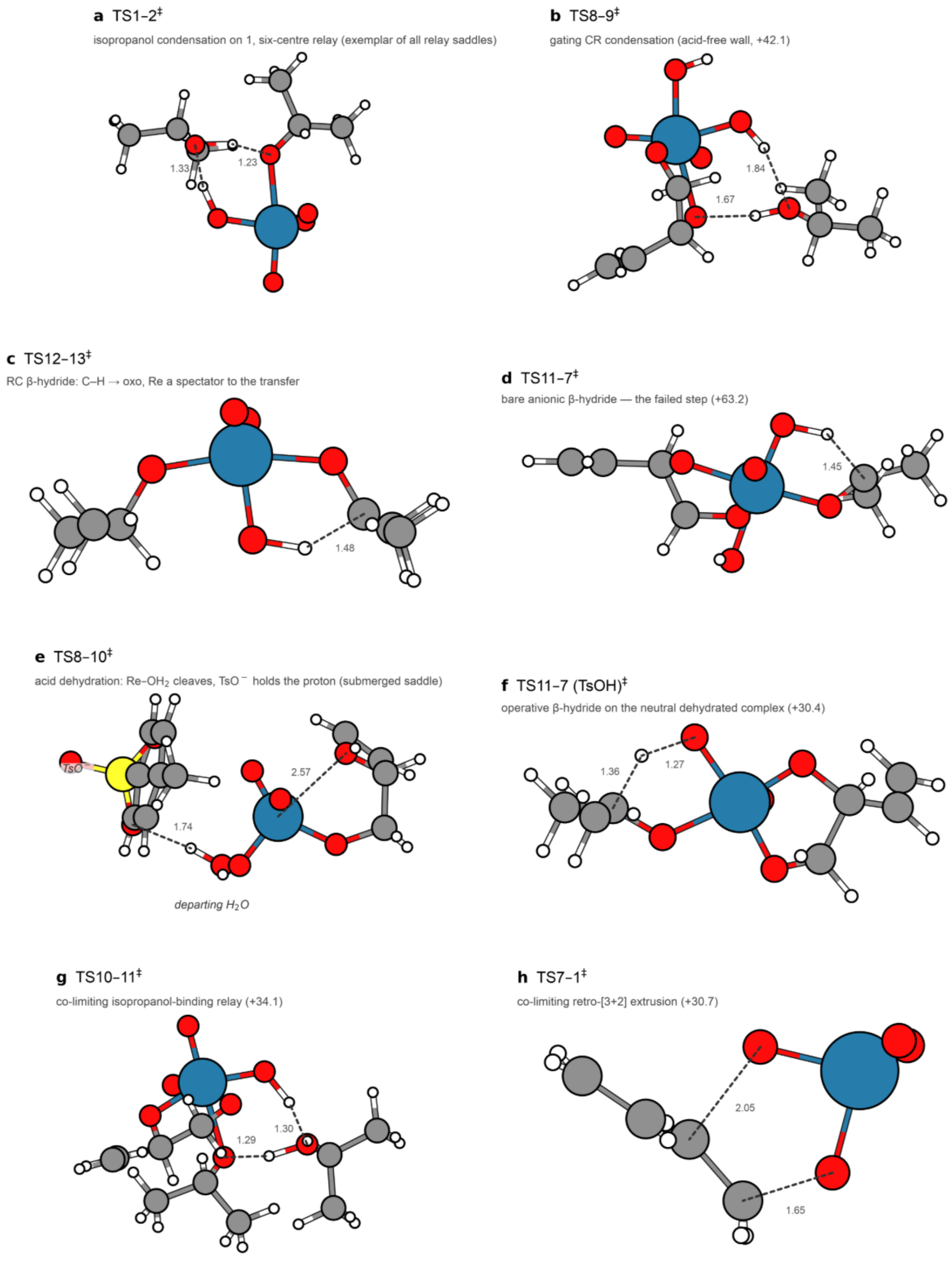

Figure S1. *Optimised geometries of the eight decision-carrying transition states. Dashed lines mark two things: partial bonds, meaning the contacts forming and breaking along the reaction coordinate, and, separately, hydrogen bonds. Both are labelled with their distances in Å.*

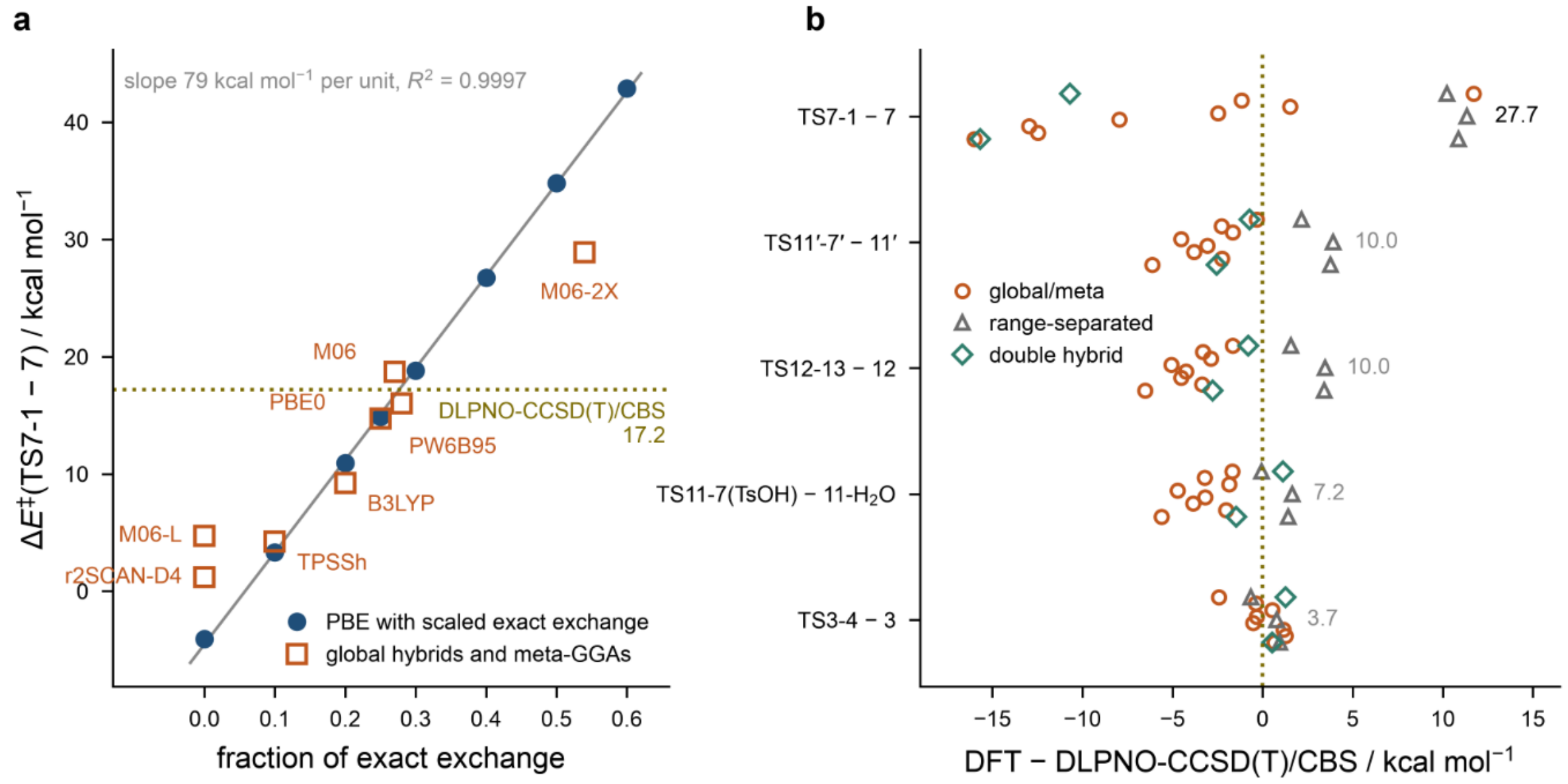

Figure S2. (a) The **TS7-1** electronic barrier (def2-TZVP, gas phase) as a function of the fraction of exact exchange: the PBE functional with scaled exact exchange (the filled circles, with a linear fit) and the global hybrids and meta-GGAs listed in Table S2 (the open squares); the dotted line represents the DLPNO-CCSD(T)/CBS value. (b) The deviation from the DLPNO-CCSD(T)/CBS value for the thirteen functionals for the five coupled-cluster-anchored barriers: the global hybrids and meta-GGAs (circles), the range-separated hybrids (triangles) and the double hybrids (diamonds); the number adjacent to each row is the range among the thirteen functionals.

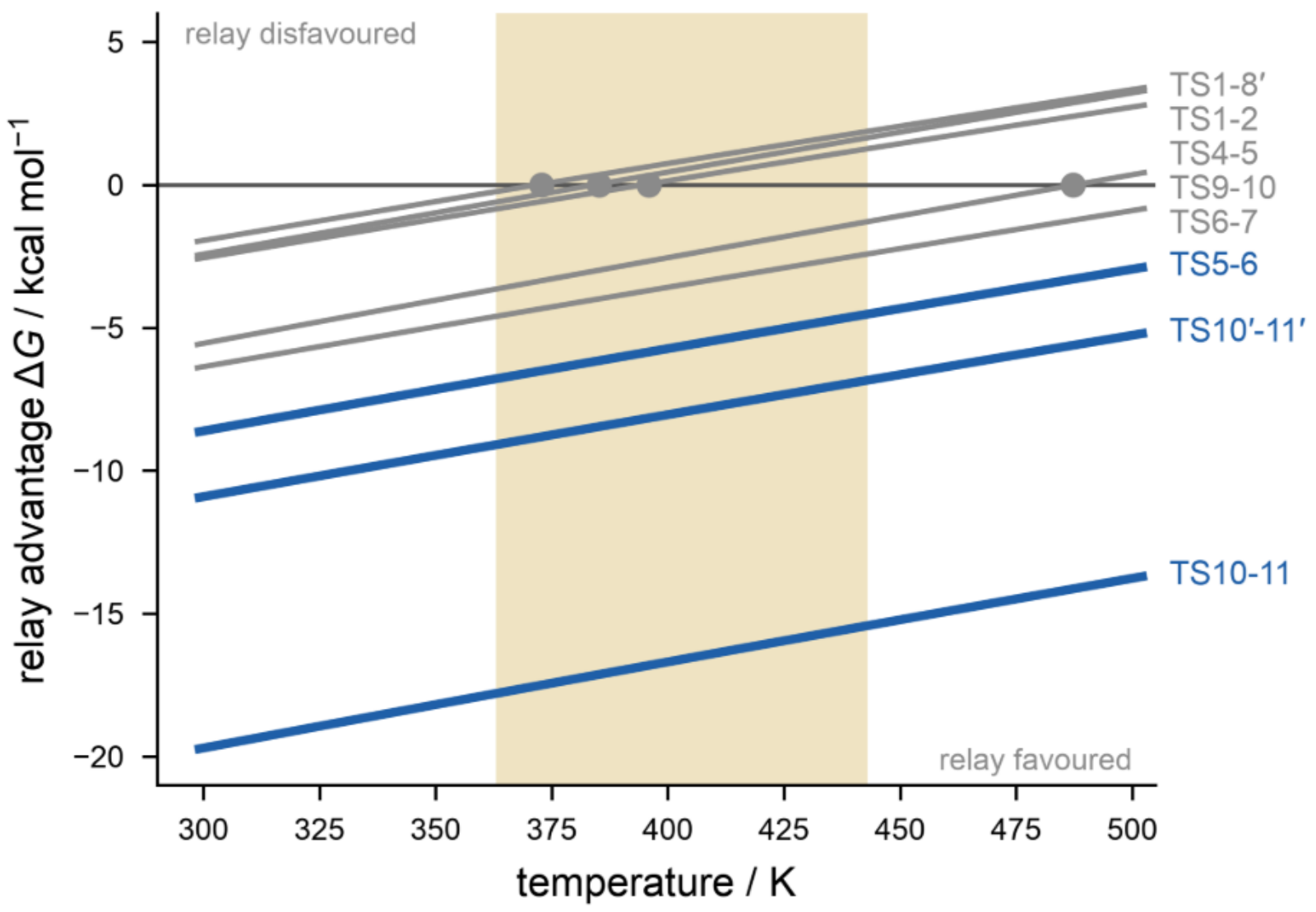

Figure S3. *Because there is a compromise between enthalpy and entropy when a first-shell solvent molecule can be involved in order to form a 6-membered ring TS (called relay TS), its activation energy depends on temperature. For the eight transitions states where a relay TS is possible, the relay advantage, Δ, is defined as the difference between ΔG(six-centre) and the sum of ΔG(four-centre) and ΔG(iPrOH), over the temperature range from 298 to 503 K. Blue: the three pairs still clearly relay-favoured at 413 K; the crossover points within the temperature range are shown by filled circles; the shaded band indicates the 363 to 443 K operating window for this reaction.*

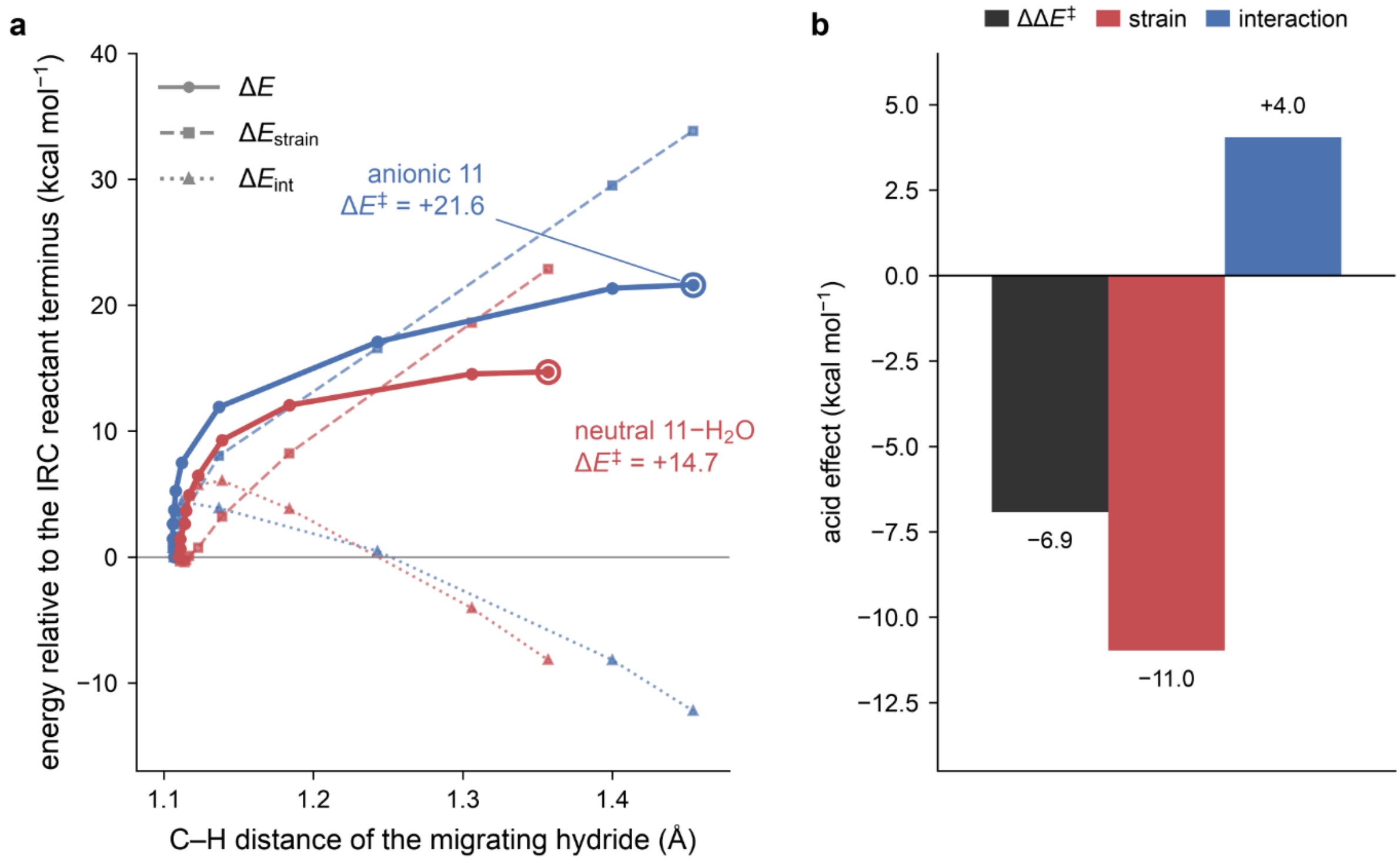

Figure S4. The origin of the reduction in the β-hydride transfer barrier (PBE0-D3(BJ)/def2-TZVP/PCM electronic energies on B3LYP geometries). (a) Activation-strain decomposition along the intrinsic reaction coordinate (IRC) for the anionic saddle ***TS11-7*** *(blue) and the neutral saddle* ***TS11^{DEHYD}-7*** *(red), with respect to the reactant end of each IRC: the total energy ΔE (solid), the strain energy ΔE_{strain} (dashed) and the interaction energy ΔE_{int} (dotted) are plotted against the C–H distance of the hydrogen that is migrating; the saddles are shown by circles. (b) The change in barrier, strain and interaction when passing from the anionic to the neutral saddle; negative values show that the acid lowers the term. The IRC endpoints do not quite match the reagents in the free energy profile, as the IRC endpoint is not always the most stable conformer; a further conformational change is left unrepresented in the free energy profiles (this explains why the strain-interaction analysis does not add up to the entire difference in activation barriers).*

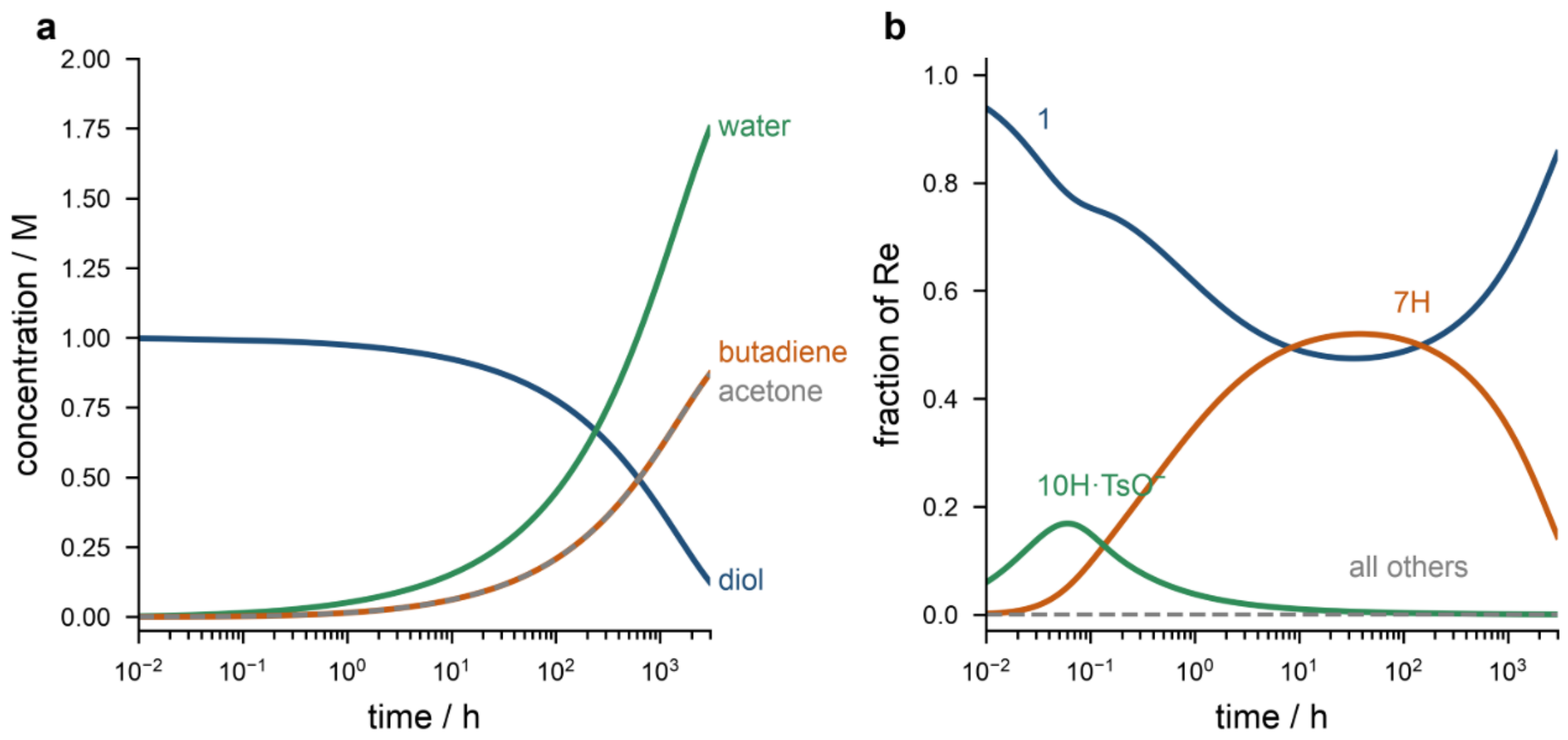

Figure S5. Concentration–time profiles of the operative microkinetic model integrated as a closed batch reactor at 413 K (composite CC level; 1.0 M but-3-ene-1,2-diol, 13 M iPrOH, 0.03 M ReO_4^-, 0.03 M TsOH; no water, acetone or butadiene at t = 0). (a) Free species: diol, butadiene, acetone (dashed; equal to butadiene by stoichiometry, both being released at or after ***7H****) and water. (b) Fraction of rhenium present as* ***1****,* ***10H****·TsO$^-$ and* ***7H****; "all others" is the sum over the remaining intermediates, none of which exceeds 10^{-5} of the rhenium. Time on a logarithmic axis.*

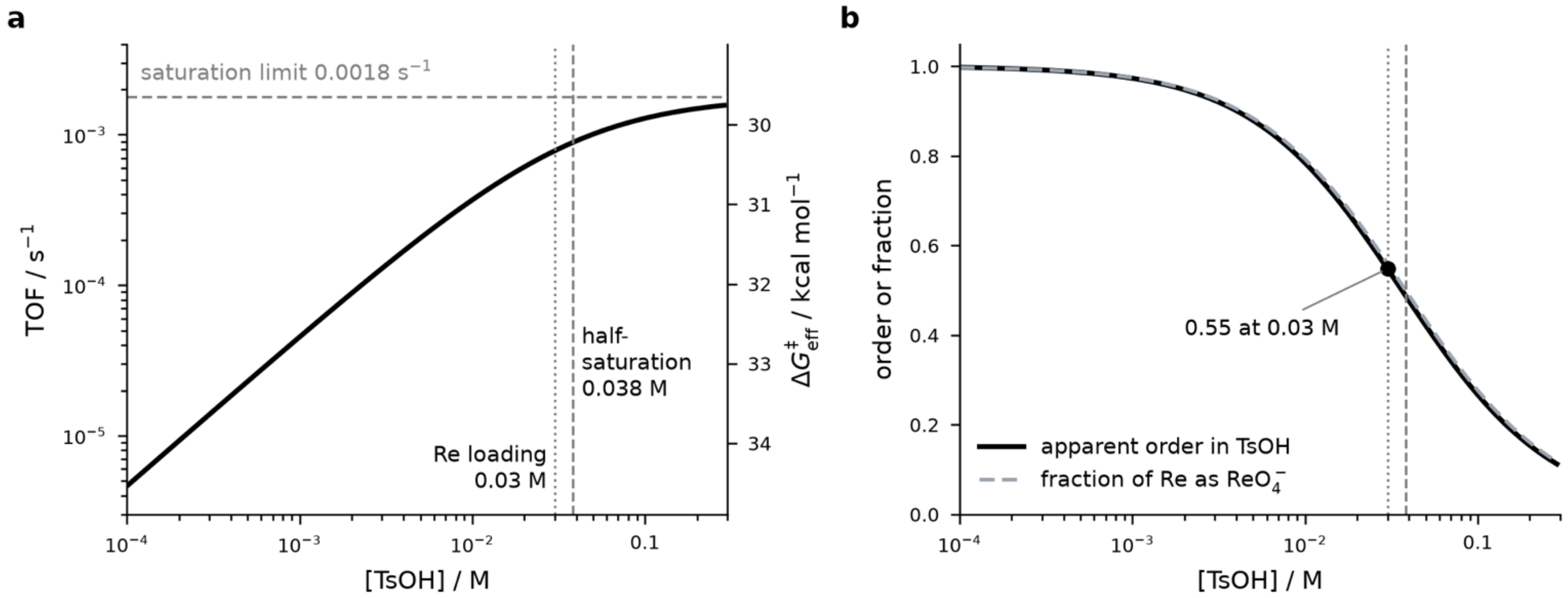

Figure S6. Acid dependence at 413 K (0.03 M ReO_4^-, 1 M diol, 13 M iPrOH; 0.1% conversion). (a) Turnover frequency against TsOH concentration; the right axis gives the equivalent Δ$G^{\ddagger}_{eff}$. Half-saturation (dashed, 0.038 M) lies next to the Re loading (dotted, 0.03 M). (b) Apparent order in TsOH and fraction of Re present as ReO_4^-.

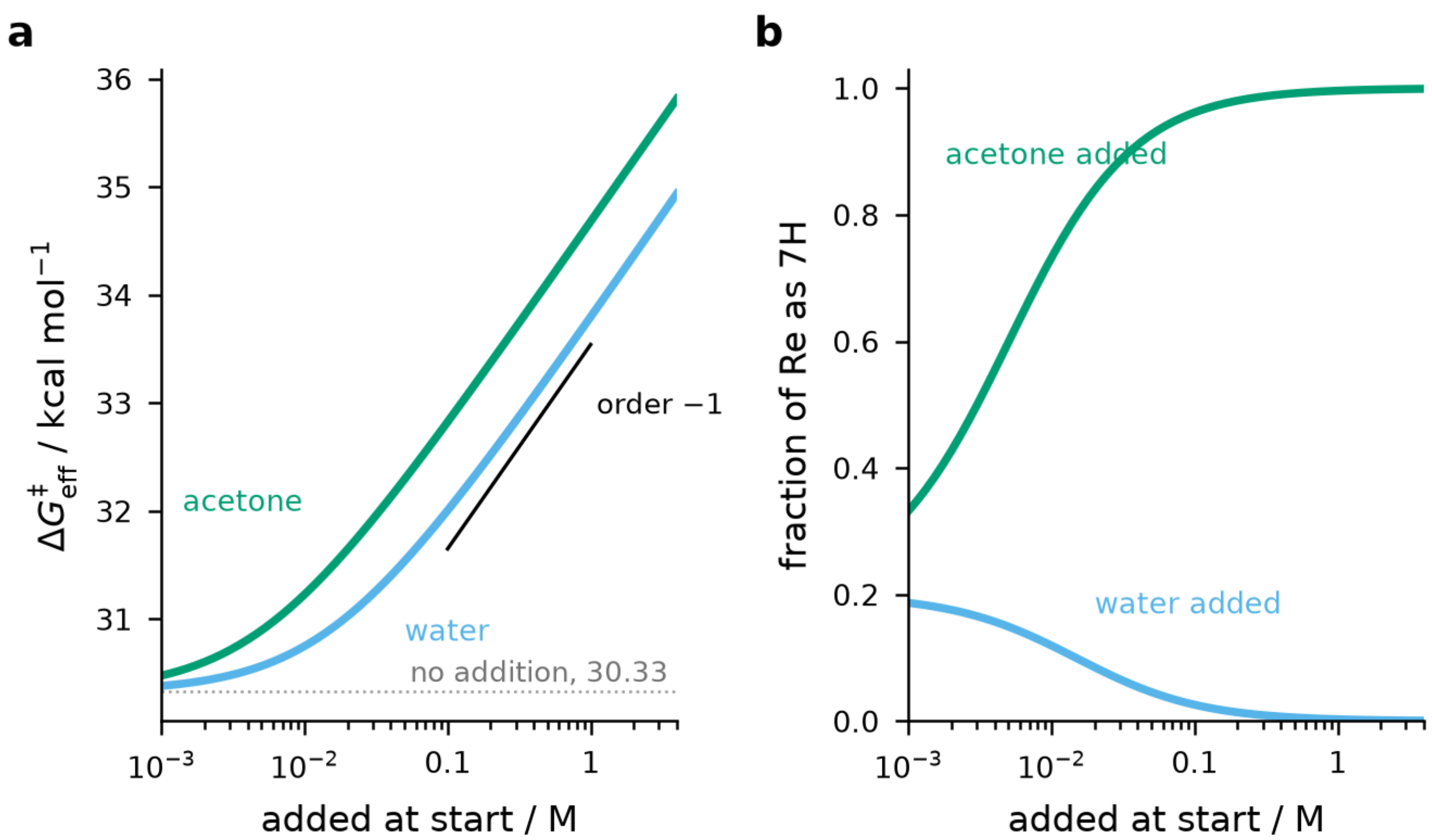

Figure S7. Co-product inhibition according to microkinetic modelling at 413 K. (a) Effective barrier at 0.1% conversion as a function of the quantity of extra water or acetone added at the start. (b) Fraction of Re as species **7H**, showing that ketone inhibition is due to retention of Re as **7H**.

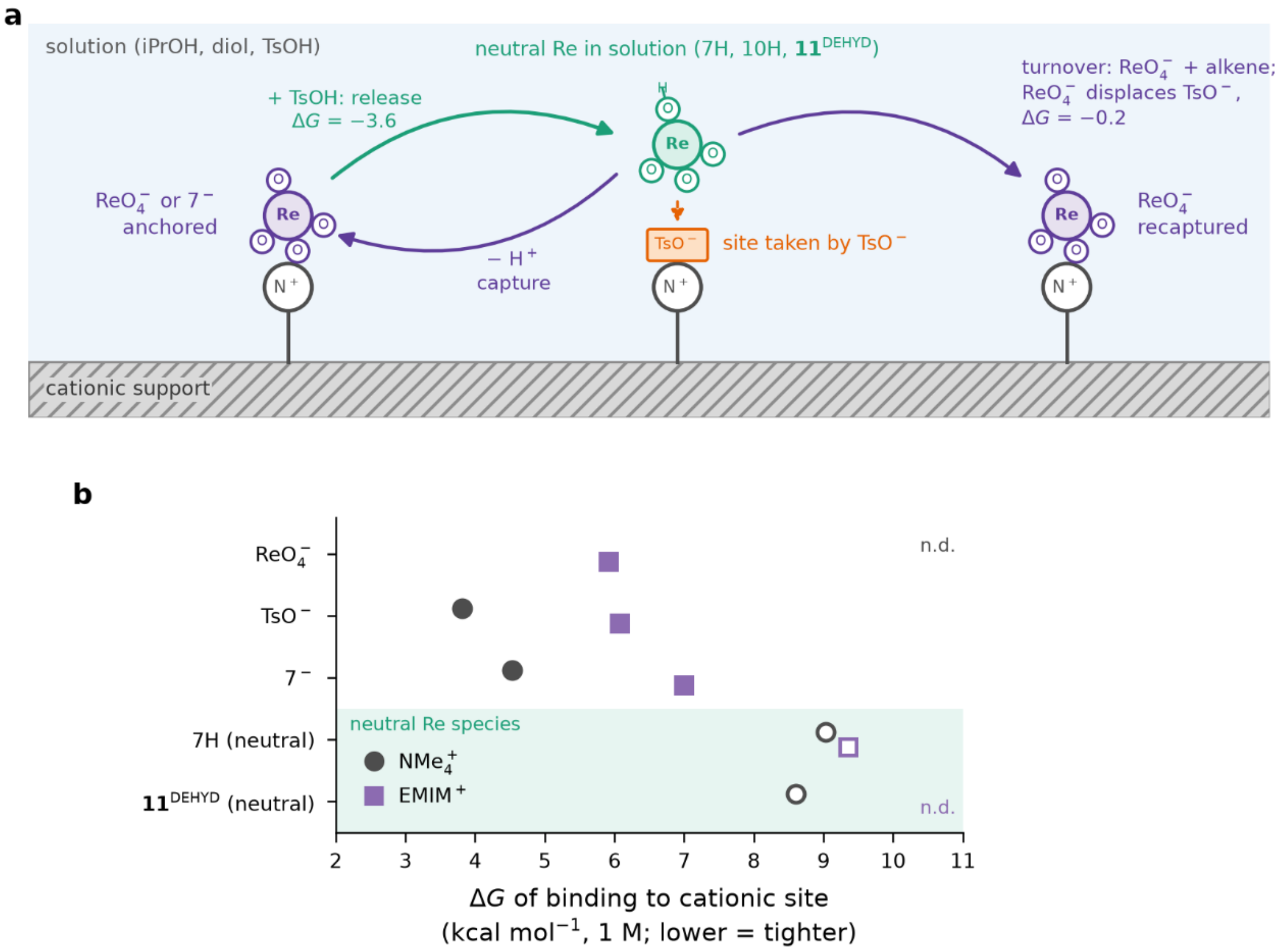

Figure S8. Schematic representation of a cation-anchored heterogeneous perrhenate catalyst. Anionic Re is electrostatically bound at the interface, whereas protonation by TsOH allows neutral Re into the solution while TsO^- becomes the electrostatically bound anion. Free energies are calculated by modelling the cation as an $EMIM^+$ (1-ethyl-3-methylimidazolium) site.

S10. References

1 M. J. Frisch, G. W. Trucks, H. B. Schlegel, G. E. Scuseria, M. A. Robb, J. R. Cheeseman, G. Scalmani, V. Barone, G. A. Petersson, H. Nakatsuji, X. Li, M. Caricato, A. V. Marenich, J. Bloino, B. G. Janesko, R. Gomperts, B. Mennucci, H. P. Hratchian, J. V. Ortiz, A. F. Izmaylov, J. L. Sonnenberg, D. Williams-Young, F. Ding, F. Lipparini, F. Egidi, J. Goings, B. Peng, A. Petrone, T. Henderson, D. Ranasinghe, V. G. Zakrzewski, J. Gao, N. Rega, G. Zheng, W. Liang, M. Hada, M. Ehara, K. Toyota, R. Fukuda, J. Hasegawa, M. Ishida, T. Nakajima, Y. Honda, O. Kitao, H. Nakai, T. Vreven, K. Throssell, J. A. Montgomery, Jr., J. E. Peralta, F. Ogliaro, M. J. Bearpark, J. J. Heyd, E. N. Brothers, K. N. Kudin, V. N. Staroverov, T. A. Keith, R. Kobayashi, J. Normand, K. Raghavachari, A. P. Rendell, J. C. Burant, S. S. Iyengar, J. Tomasi, M. Cossi, J. M. Millam, M. Klene, C. Adamo, R. Cammi, J. W. Ochterski, R. L. Martin, K. Morokuma, O. Farkas, J. B. Foresman, and D. J. Fox, GAUSSIAN 16 (Revision C.01), Gaussian Inc., Wallingford, CT, 2019.

2 A. D. Becke, *J. Chem. Phys.*, 1993, 98, 5648; C. Lee, W. Yang and R. G. Parr, *Phys. Rev. B*, 1988, 37, 785; P. J. Stephens, F. J. Devlin, C. F. Chabalowski and M. J. Frisch, *J. Phys. Chem.*, 1994, 98, 11623.

3 S. Grimme, J. Antony, S. Ehrlich and H. Krieg, J. Chem. Phys., 2010, 132, 154104; S. Grimme, S. Ehrlich and L. Goerigk, *J. Comput. Chem.*, 2011, 32, 1456.

4 F. Weigend and R. Ahlrichs, *Phys. Chem. Chem. Phys.*, 2005, 7, 3297.

5 D. Andrae, U. Häussermann, M. Dolg, H. Stoll and H. Preuss, *Theor. Chim. Acta*, 1990, 77, 123.

6 J. Tomasi, B. Mennucci and R. Cammi, *Chem. Rev.*, 2005, 105, 2999; G. Scalmani and M. J. Frisch, *J. Chem. Phys.*, 2010, 132, 114110.

7 K. Fukui, Acc. Chem. Res., 1981, 14, 363; H. P. Hratchian and H. B. Schlegel, *J. Chem. Theory Comput.*, 2005, 1, 61.

8 G. Luchini, J. V. Alegre-Requena, I. Funes-Ardoiz and R. S. Paton, *F1000Research*, 2020, 9, 291.

9 R. F. Ribeiro, A. V. Marenich, C. J. Cramer and D. G. Truhlar, *J. Phys. Chem. B*, 2011, 115, 14556.

10 E. Wigner, Z. *Phys. Chem. B*, 1932, 19, 203.

11 R. T. Skodje and D. G. Truhlar, *J. Phys. Chem.*, 1981, 85, 624.

12 P. M. Zimmerman, *J. Chem. Phys.*, 2013, 138, 184102.

13 C. Adamo and V. Barone, *J. Chem. Phys.*, 1999, 110, 6158.

14 C. Riplinger and F. Neese, *J. Chem. Phys.*, 2013, 138, 034106; C. Riplinger, P. Pinski, U. Becker, E. F. Valeev and F. Neese, *J. Chem. Phys.*, 2016, 144, 024109.

15 F. Neese and E. F. Valeev, *J. Chem. Theory Comput.*, 2011, 7, 33.

16 F. Neese, *WIREs Comput. Mol. Sci.*, 2022, 12, e1606.

17 Functionals used in the level-of-theory benchmark of Section S2: Y. Zhao and D. G. Truhlar, Theor. Chem. Acc., 2008, 120, 215; Y. Zhao and D. G. Truhlar, *J. Chem. Phys.*, 2006, 125, 194101; N. Mardirossian and M. Head-Gordon, Phys. Chem. Chem. Phys., 2014, 16, 9904; N. Mardirossian and M. Head-Gordon, *J. Chem. Phys.*, 2016, 144, 214110; Y.-S. Lin, G.-D. Li, S.-P. Mao and J.-D. Chai, *J. Chem. Theory Comput.*, 2013, 9, 263; Y. Zhao and D. G. Truhlar, J. Phys. Chem. A, 2005, 109, 5656; V. N. Staroverov, G. E. Scuseria, J. Tao and J. P. Perdew, J. Chem. Phys., 2003, 119, 12129; S. Grimme, *J. Chem. Phys.*, 2006, 124, 034108; G. Santra, N. Sylvetsky and J. M. L. Martin, *J. Phys. Chem. A*, 2019, 123, 5129; J. W. Furness, A. D. Kaplan, J. Ning, J. P. Perdew and J. Sun, *J. Phys. Chem. Lett.*, 2020, 11, 8208; E. Caldeweyher, S. Ehlert, A. Hansen, H. Neugebauer, S. Spicher, C. Bannwarth and S. Grimme, *J. Chem. Phys.*, 2019, 150, 154122.

18 A. J. Cohen, P. Mori-Sánchez and W. Yang, *Science*, 2008, 321, 792.

19 T. J. Lee and P. R. Taylor, *Int. J. Quantum Chem.*, 1989, 36, 199.

20 W. Jiang, N. J. DeYonker and A. K. Wilson, *J. Chem. Theory Comput.*, 2012, 8, 460.

21 P. Pracht, F. Bohle and S. Grimme, *Phys. Chem. Chem. Phys.*, 2020, 22, 7169.

22 C. Bannwarth, S. Ehlert and S. Grimme, *J. Chem. Theory Comput.*, 2019, 15, 1652.

23 A. E. Reed, L. A. Curtiss and F. Weinhold, *Chem. Rev.*, 1988, 88, 899.

24 K. B. Wiberg, *Tetrahedron*, 1968, 24, 1083.

25 F. M. Bickelhaupt and K. N. Houk, *Angew. Chem. Int. Ed.*, 2017, 56, 10070.

26 C. T. Campbell, ACS Catal., 2017, 7, 2770; C. Stegelmann, A. Andreasen and C. T. Campbell, *J. Am. Chem. Soc.*, 2009, 131, 8077.

27 E. N. Szlapa and J. N. Harvey, *Chem. Eur. J.*, 2018, 24, 17096.

28 M. Besora, J.-L. Carreón-Macedo, A. J. Cowan, M. W. George, J. N. Harvey, P. Portius, K. L. Ronayne, X.-Z. Sun and M. Towrie, *J. Am. Chem. Soc.*, 2009, 131, 3583.

29 C. Boucher-Jacobs and K. M. Nicholas, *ChemSusChem*, 2013, 6, 597.

30 I. Ahmad, G. Chapman and K. M. Nicholas, *Organometallics*, 2011, 30, 2810.

31 S. Liu, A. Senocak, J. L. Smeltz, L. Yang, B. Wegenhart, J. Yi, H. I. Kenttämaa, E. A. Ison and M. M. Abu-Omar, *Organometallics*, 2013, 32, 3210.

32 X. Li and Y. Zhang, ChemSusChem, 2016, 9, 2774.

33 S. Doherty, J. G. Knight, M. A. Carroll, J. R. Ellison, S. J. Hobson, S. Stevens, C. Hardacre and P. Goodrich, Green Chem., 2015, 17, 1559.

34 K. Kuwamoto, M. Okamura and S. Hikichi, ChemistryEurope, 2024, 2, e202300080.
